# Protein expression reveals a molecular sexual identity of avian primordial germ cells at pre-gonadal stages

**DOI:** 10.1038/s41598-021-98454-2

**Published:** 2021-09-28

**Authors:** Laura Soler, Sabine Alves, Aurélien Brionne, Aurore Jacques, Vanessa Guérin, Maeva Cherif-Feildel, Lucie Combes-Soia, Sophie Fouchécourt, Aurore Thélie, Elisabeth Blesbois, Michael J. McGrew, Valérie Labas, Marina S. Govoroun

**Affiliations:** 1 INRAE, CNRS, Université de Tours, IFCE, PRC, 37380 Nouzilly, France; 2 Present Address: Toxalim (Research Centre in Food Toxicology), INRAE, Université de Toulouse, ENVT, INP-Purpan, UPS, 31027 Toulouse, France; 3 INRAE, Université de Tours, BOA, 37380 Nouzilly, France; 4 INRAE, CHU de Tours, Université de Tours, PRC, PIXANIM, 37380 Nouzilly, France; 5 The Roslin Institute and Royal (Dick) School of Veterinary Studies, University of Edinburgh, Easter Bush Campus, Midlothian, EH25 9RG UK

**Keywords:** Developmental biology, Genetics, Stem cells

## Abstract

In poultry, in vitro propagated primordial germ cells (PGCs) represent an important tool for the cryopreservation of avian genetic resources. However, several studies have highlighted sexual differences exhibited by PGCs during in vitro propagation, which may compromise their reproductive capacities. To understand this phenomenon, we compared the proteome of pregonadal migratory male (ZZ) and female (ZW) chicken PGCs propagated in vitro by quantitative proteomic analysis using a GeLC-MS/MS strategy. Many proteins were found to be differentially abundant in chicken male and female PGCs indicating their early sexual identity. Many of the proteins more highly expressed in male PGCs were encoded by genes localised to the Z sex chromosome. This suggests that the known lack of dosage compensation of the transcription of Z-linked genes between sexes persists at the protein level in PGCs, and that this may be a key factor of their autonomous sex differentiation. We also found that globally, protein differences do not closely correlate with transcript differences indicating a selective translational mechanism in PGCs. Male and female PGC expressed protein sets were associated with differential biological processes and contained proteins known to be biologically relevant for male and female germ cell development, respectively. We also discovered that female PGCs have a higher capacity to uptake proteins from the cell culture medium than male PGCs. This study presents the first evidence of an early predetermined sex specific cell fate of chicken PGCs and their sexual molecular specificities which will enable the development of more precise sex-specific in vitro culture conditions for the preservation of avian genetic resources.

## Introduction

The protection of endangered avian breeds requires the preservation of the entire genotype of the animal. Bird oocytes and zygotes are very lipid rich, which hinders their cryopreservation. For this reason, the conservation of avian genetic resources is essentially based on semen cryopreservation. Semen-based breed recovery, however, is not optimal as it requires many successive generations of insemination to restore the breed genotype and does not conserve the female specific W sex chromosome^1^. Hence, there is a need to develop new reproductive technologies that allow the collection, propagation and preservation of avian reproductive germ cells.

The precursors of the germ line are called primordial germ cells (PGCs). PGCs are embryonic unipotent stem cells fated to develop into gametes in the appropriate cellular and molecular environment. Aves is one of the few animal classes from which PGCs can be isolated at early embryonic stages due to their precocious formation in avian species and the particularities of their migration through the avian embryo. Avian PGCs can be isolated at pre-migratory stages from the embryonic germinal crescent, during their migration in the gonads by blood sampling, or from the gonads before the onset of their sex differentiation^2,3^.

Isolated PGCs from the chicken, *Gallus gallus*, can be propagated indefinitely in vitro, cryopreserved and/or transplanted into host embryos, where they will develop into functional gametes^4–6^. In poultry, the development of these methods has advanced the use of PGCs for the conservation of endangered indigenous breeds and of valuable commercial genetic resources as well as offering an efficient vehicle for genome editing and transgenesis^6–8^.

In the animal kingdom, PGCs show some degree of sexual plasticity, which is inversely correlated with their level of complexity. In molluscs, fish, amphibians and other animals with poorly differentiated sex chromosomes, male and female PGCs are sexually bipotential and may develop into spermatozoa or ova depending on hormonal and cellular contexts regulated by the environment^9^. In animals with highly differentiated X and Y sex chromosomes, such as mammals, the differentiation of male and female PGCs into functional gametes is highly restricted by chromosomal factors^10^. However, several experiments carried out in rodents have shown that PGCs can have some sexual plasticity. In some natural genetic contexts or in experimentally created sexual reversals, XY germ cells may develop into functional oocytes although with a considerable decrease in fertility^11^. In contrast, XX germ cells never develop into spermatozoa, the second X chromosome being incompatible with spermatogenesis^10^. In addition, some Y-linked genes are required for the resumption of second meiotic division in spermatocytes^12^.

Birds have a ZZ/ZW system where the female is heterogametic (ZW), and male is homogametic (ZZ). The sex chromosomes Z and W are well differentiated, however, the existing knowledge on the sexual plasticity of avian PGCs is still rudimental and contradictory. Studies on chicken germline intersex chimeras obtained after the direct transplantation of donor PGCs to recipient embryos of the opposite sex suggest that chicken germ lineage development is under the strict control of genetic and epigenetic factors, but might have some degree of sexual plasticity. After the direct transplantation following PGC isolation of a ZZ male donor/ZW female recipient combination, progeny from donor-derived cells is very rare event. No germline transmission is generally found in the case of the reverse ZW female donor/ZZ male recipient combination^13^. Similarly, no donor-derived progeny were observed after intersex transplantation of ZZ or ZW PGCs*,* when they have been propagated in vitro before transplantation^6,14–16^. Moreover, *in ovo* treatment of female embryos with aromatase inhibitor induces a female-to-male sex reversal, but these animals produce non-functional spermatozoa^17,18^. However, ZZ donor PGCs propagated in vitro were observed to differentiate into functional oocytes and produce progeny after transplantation to partially sterilised ZW host embryo^19^. These data suggest that chicken PGC developmental plasticity may depend on several factors including hormonal environment, the host biological context and in vitro culture conditions, which can reveal sex-specific features of these cells. Indeed, survival and propagation of chicken PGCs in vitro is sex-dependent^3,14^ and their reproductive capacity may be compromised in a sex specific manner during in vitro propagation^4,5^. However, the molecular nature of this phenomenon is largely unknown.

The objective of the present study is to provide for the first time a description of the intrinsic differences of the molecular phenotypes of male (ZZ) and female (ZW) chicken PGCs isolated and propagated in vitro*,* using media conditions that support the proliferation of PGCs from both sexes^5^. We investigated the protein differences of ZZ and ZW chicken PGCs by a quantitative bottom-up proteomic approach using a GeLC-MS/MS strategy, followed by a functional analysis of the data. This allowed us to identify and validate differential biologically relevant pathways and cellular functions, pointing to an early sexual molecular identity of ZZ and ZW PGCs.

## Methods

### Ethical statement

The housing and the breeding procedures to obtain eggs were approved by local official ethics committee (Comité d’Ethique en Expérimentation Animale Val de Loire CEEA – n°19), by the French Ministry of Agriculture and French Veterinary Services regulations under agreement number C37-175–1. The experiments were performed in accordance to European welfare regulations (European directive 2010/63/EU).

### Animals

Animals were housed at the INRA experimental facility PEAT: https://doi.org/10.15454/1.5572326250887292E12 (Nouzilly, France). Twenty weeks old male and female chickens of local breed Noire du Berry were obtained from the Club Français de la Poule Noire du Berry. This local breed was used as it is part of a Genetics Resource conservation program developed for French rare endangered breeds. Males were housed in individual battery cages and females in groups of 5 under 14L/10D photoperiod and fed with a standard diet of 12.5 MegaJoules/day supplemented with calcium for females. For fertile egg production, 20-weeks old hens were fertilized by artificial insemination using pooled sperm from 5 males that was collected twice a week by massage^20^. Eggs were incubated at 37.7° C and 45–51% humidity for 56 h with turning over an angle of 45° either side of the short axis of the egg.

### Culture derivation and preparation of samples for further analyses

After 56 h of incubation, a small window was made in the egg shell of the pointed end of the egg and 1.0–2.0 µls of blood containing circulating PGCs were aspirated from dorsal aorta using fine glass capillary and placed immediately in culture medium. The culture media and culture conditions were set up as described in^5^ with slight modifications: instead of using Activin A, or Activin A and BMP4, the culture medium was supplemented with 25 ng/ml of BMP4 only. Five male and five female PGCs cultures deriving from individual embryos were in vitro expanded for 1.5–2 months until each culture population reached 30 million cells. For protein extraction, 2.0 × 10^6^ cells were centrifuged at 1000 g for 10 min, washed twice with 50 volumes of Mg^2+^ and Ca^2+^ free PBS buffer and frozen immediately in liquid nitrogen. For RNA extraction 5.0 × 10^5^ cells were centrifuged at 1000 g for 10 min, then resuspended in lysis buffer of NucleoSpin RNA XS kit (MACHEREY–NAGEL, France) and frozen immediately in liquid nitrogen. All samples were stored at − 80 °C until proceeding with protein or RNA extraction.

### Sexing of embryos and cells

Sexing of embryos and PGCs was performed by PCR as described previously^21–23^. For DNA extraction from PGCs, 60,000 cultured PGCs were incubated in 20 µl of proteinase K 0.25 mg/ml water solution at 55 °C for 1 h. After inactivation of proteinase K at 99° C for 10 min, DNA were stored at 4 °C for further analysis.

### Protein extraction and SDS-PAGE

Proteins were extracted from samples containing 2.0 × 10^6^ PGCs in 250 µl of 6 M Urea, 4% SDS, 50 mM pH 8.8 Tris–HCl containing protease inhibitors (Roche diagnostic, Paris, France) by sonication on ice. Extracted proteins were allowed to solubilize for 20 min at room temperature under shaking. After centrifugation at 10,000 g for 10 min, protein concentration was quantified from protein extracts (Thermo Scientific Pierce BCA Protein Assay Kit). Male and female PGCs protein extract pools were prepared by combining 10 µg of total protein from each sample. Fifty µg of each pool were reduced using DTT 20 mg/ml and denatured at 95 °C for 5 min before being loaded in a 4–12% SDS-PAGE gel. The gel was run at 30 mA until full migration of the bromophenol blue front.

### In-gel protein digestion and NanoLC-MS/MS for PGC protein identification

After separation of proteins by SDS-PAGE and Coomassie Blue staining, each lane was cut into 10 slices. After washing of gel slices, proteins were reduced, alkylated and in-gel digested by trypsin, as described in Supplementary Methods. For each protein band, the resultant peptide mixture was analysed by nanoflow liquid chromatography tandem mass spectrometry (nanoLC-MS/MS) in triplicate. MS/MS ion searches were performed using Mascot search engine v 2.3 (Matrix Science, London, UK) and confronted against the “chordata” section of a locally maintained copy of nr NCBI (download Jan. 2018, 9 822 753 sequences). After identification, proteins were validated using Scaffold software (v 4.8, Proteome Software, Portland, USA).

### Label-free protein quantification

For comparative analysis, we employed Scaffold Q + software (version 4.8.4, Proteome Software, Portland, USA) to apply two independent quantitative methods: (1) the Spectral Counting (SC) using the “Weighed Spectra” option; (2) the Average Precursor Intensity (API). Thus, values of Normalized Weighed Spectra (NWS) and Normalized Average Precursor Intensity (NAPI) were tabulated using experiment wide protein clustering. Statistical analyses were performed using T-tests, where *p* < 0.05 was considered significant. Limits of an average normalized weighted spectra (NWS) ≥ 3 and male/female fold-change (FC) ≥ 2 and < 0.5 were used to increase validity.

### Bioinformatic analysis

Biological interpretations were carried out using Gene Ontology (GO) public database with the use of Biological process (BP) and Molecular Function (MF) categories using VISEAGO R package^24^. Associated Gene terms, from the identified *Gallus gallus* proteins, were retrieved from EnterGene^25^ and well-annotated species orthologues. Enrichment tests were performed for each studied comparison. All enriched GO terms (*p* < 0.01) were grouped into functional clusters using hierarchical clustering based on Wang’s semantic similarity between GO terms respecting GO graph topology and Ward’s criterion.

Chromosome enrichment tests were performed for each cluster independently using Fisher's exact test (*p*-value < 0.01) using R version 3.6.1.

For proteins for which automatic analysis assigned their corresponding genes to sex-chromosomes Z or W, blast P and TblastN^26^ were performed individually in Gallus gallus Ensembl genome browser^27^ and NCBI^28^ in order to search for their counterparts on the second sex chromosome.

### Western blot

After PGC protein extraction, proteins were separated in 10% SDS-PAGE and blotted onto nitrocellulose membranes. To evaluate hydroxysteroid dehydrogenase like 2 (HSDL2), ovalbumin and insulin-like growth factor 2 binding protein 1 (IGF2BP1) abundance rabbit anti-human HSDL2 antibody HPA050453(1:500), rabbit anti-chicken egg albumin antibody C6534 (1:500) (Sigma Aldrich, France) mouse anti-human IGF2BP1antibody PCRP-IGF2BP1-2D4 (DSHB, Iowa, USA) (1:50) and secondary goat anti-rabbit or goat anti-mouse IRDy e800CW secondary antibody (LI-COR Biosciences—GmbH) were used for HSDL2 and Ovalbumin, and IGF2BP1 respectively. Total protein staining of the membrane with SyproRuby blot stain (ThermoFischer) or Revert 700 Total Protein Stain (Li-Cor LI-COR Biosciences—GmbH) prior to blocking served as a loading control. For each lane, band intensity values were normalized onto the respective overall protein staining. Detailed procedure is described in Supplementary Mat&Met. Statistical analysis was performed by Fisher's F-test to compare two variances, followed by Student’s T-test to reveal significant differences. Differences were considered significant when *p* < 0.05.

### Reverse transcription quantitative real time polymerase chain reaction (RT-QPCR)

Total RNA was isolated from individual PGCs cultures using NucleoSpin RNA XS kit (MACHEREY–NAGEL, France) and was reverse transcribed (RT) using Thermo Scientific Maxima First Strand cDNA Synthesis Kit for RT-qPCR (ThermoFisher Scientific, France) according manufacture instructions. RT-qPCR was performed using SsoAdvanced Universal SYBR Green Supermix (BIORAD, France) according manufactory instructions. Three technical replicates were performed for each reaction. At least two independent qPCR experiments were carried out for each sample and for each primer pair. The sequences of primers used for cDNAs amplifications are presented in Supplementary Table S1. Efficacy of each primer pairs was between 1.8 and 2.2. The relative amount of transcript expression was calculated by LightCycler 480 software release 1.5.0 using standard curve composed of serial dilutions of the cDNA obtained by RT of total RNA extracted from the pool of PGCs containing equal amounts of five ZZ and five ZW PGCs cultures used for proteomic analysis. RT-qPCR products were verified by sequencing at Genewiz (Germany). For cultured PGCs characterisation the relative transcript expression level of target genes was normalized by the mean of expression ratios of two housekeeping genes: eukaryotic translation elongation factor 1 alpha 1 (EEF1A) and ribosomal protein L15 (RPL15). For evaluation of the expression of transcripts corresponding to differentially abundant proteins four housekeeping genes were used: EEF1A, actin, beta (ACTB), glyceraldehyde-3-phosphate dehydrogenase (GAPDH), RPL15. Statistical analysis was performed by Fisher's F-test to compare two variances, followed by Student’s T-test to reveal significant differences. Differences were considered significant when *p* < 0.05.

### Immunohistochemistry

After fixation and permeabilization on glass slides, cells were incubated with rabbit anti chicken DDX4 antibodies (1:500) kindly provided by B. Pain^29^ or with mouse anti-mouse SSEA1 monoclonal antibody (DSHB, Iowa, USA) (1:40). After washing cells were incubated with corresponding cross-Adsorbed Alexa Fluor secondary antibodies. For negative control Rabbit IgG or Mouse IgG (Invitrogen) were used as primary antibodies for DDX4 or SSEA1 analysis, respectively. Detailed procedure is described in supplementary Mat&Met.

### Gonad colonisation test

The PGCs were labelled with green fluorescent dye PKH67 (SIGMA-Aldrich, France) according manufacture instructions. 1.0 µl of culture medium containing 2000 labelled PGCs was injected into the embryonic dorsal aorta through the small window in the eggshell of the egg incubated for 56 h. The shell window was sealed with Parafilm M (PRAXISDIENST, France) and the eggs were incubated for 90 additional hours. The embryos were removed from the egg, euthanized by decapitation and the gonad-mesonephros complexes were isolated. Green fluorescence was observed using a Zeiss Axiovert 200 microscope (Zeiss, France) microscope and the images were captured by Axiocam type MRm camera and analysed using AxioVision software (Zeiss, France).

## Results and discussion

### Proteomic analysis reveals sexual differences in chicken PGCs

Here, for the first-time, we conduct a comparative proteomic analysis of functional male and female PGCs propagated in vitro. Prior to the proteomics analysis, the basic molecular phenotype of the cultured male and female PGCs was confirmed using RT-qPCR analysis of selected key genes and immunohistochemistry (Supplementary Fig. S1a, b). As expected, male and female PGCs showed no difference in the expression of genes that are specific for germ cells (Deleted In Azoospermia Like; *DAZL*) and pluripotency factors (SRY-Box Transcription Factor 2; *SOX2*, Nanog Homeobox; *NANOG*) (Supplementary Fig. S1a).

A male molecular phenotype was confirmed in male PGCs by the expression level of the germ cell specific gene, *DDX4*, located in the Z-chromosome. Unlike mammals, birds do not fully compensate for lower gene expression of the Z chromosome genes in females to equalise expression in both sexes. This results in a significantly lower global expression in females (ZW) of Z-linked genes in all somatic cells^30^. According to the lack of compensation of double dosage of Z-linked chromosomal genes in birds, we expected *DDX4* to be two times more highly expressed in male (ZZ) PGCs than in female (ZW) PGCs. Our gene expression results are in agreement with the double dosage of the Z -linked gene *DEAD-Box Helicase 4* (*DDX4)* in males and with the absence of general mechanism of dosage compensation of Z-linked genes in the chicken. Immunohistochemistry analysis confirmed the presence of the germ cell-specific proteins DDX4 and stage-specific embryonic antigen-1 (SSEA-1) in PGCs from both sexes (Supplementary Fig. S1b). Additionally, we confirmed that in vitro propagated PGCs conserved their basic ability to colonize the embryonic gonads when injected in the dorsal aorta of recipient chicken embryos (Supplementary Fig. S1c).

GeLC-MS/MS analysis identified 1332 proteins with unique accession numbers distributed in 777 protein clusters (Supplementary Table S2a, b). Among these proteins, 299 were differentially abundant (*p*-value < 0.05 and male/female (M/F) FC limit 2 < FC or FC < 0,5) in male and female PGCs as determined by at least one of two used quantification methods: WS (columns G to N) and/or API (columns O to V). Among these 299 differentially abundant proteins with unique accession numbers, 154 were preferentially abundant in male PGCs and 155 were more abundant in female PGCs (Supplementary Table S2c, d). Among 154 proteins preferentially abundant in male PGCs, 32 proteins corresponding to 28 gene IDs were quantified by both WS and API, 90 proteins corresponding to 72 gene IDs were quantified by WS only and 32 proteins corresponding to 26 gene IDs by API only. Among 155 proteins preferentially abundant in female PGCs, 41 proteins corresponding to 30 gene IDs were quantified by both WS and API, 97 proteins corresponding to 78 gene IDs were quantified by WS only and 17 proteins corresponding to 16 gene IDs by API only. Both quantification methods were, thus, complementary. Out of the top 20 differentially abundant proteins (Table 1), 14 were identified only in female PGCs and 11 only in male PGCs.

**Table 1:** Top forty differentially abundant proteins in male and female PGCs identified by two quantitative methods: WS and API.

NCBI					Normalized weighed spectra			Normalized precursor intensity		
					Male/Female	Average		Male/Female	Average	
Protein accession number	Gene ID	Gene Name	Chr	Description	T-Test	Male	Female	T-Test	Male	Female
NP_001006398.1	421348	EPRS	3	Multifunctional aminoacyl-tRNA synthetase that catalyzes the aminoacylation of glutamic acid and proline tRNA species	0.00028	10. 58	0.00	0.006	4.24E + 06	8. 55E-01
NP_989854.1	395194	TLN1	Z	Cytoskeletal protein concentrated in areas of cell-substratum and cell–cell contacts. Plays a significant role in the assembly of actin filaments and in spreading and migration of various cell types	0.0094	9.06	0.00	0.0083	3.34E + 06	0.00
XP_021238991.1	110391426	HUWE1	unplaced	Functions as an E3 ubiquitin ligase required for the ubiquitination and subsequent degradation of the anti-apoptotic protein, the p53 tumor suppressor, core histones, and DNA polymerase beta	0.0059	10.57	0.00	0.0014	5.07E + 06	5.52E-01
XP_005533424.1	107050871	LOC107050871	33	DNA-dependent RNA polymerase that catalyzes the transcription of DNA into RNA using the four ribonucleoside triphosphates as substrates	0.015	13.54	0.00	0.00015	5.39E + 06	5.99E-01
XP_418787.2	420688	DNAJC13	2	Member of the Dnaj protein family whose members act as co-chaperones of a partner heat-shock protein by binding to the latter and stimulating ATP hydrolysis	0.00058	8.42	0.00	0.00045	4.58E + 06	8.55E-01
XP_004940443.1	42,811	MDN1	3	Nuclear chaperone required for maturation and nuclear export of pre-60S ribosome subunits	0.017	4.39	0.00	0.00039	5.02E + 06	8.55E-01
XP_004949443.1	427114	TTC37	Z	Protein rich in tetratricopeptide repeats, which mediate protein–protein interactions and chaperone activity	0.00096	2.91	0.00	0.00015	2.53E + 06	5.58E-01
NP_001026553.1	426429	OGDH	22	Catalyzes the overall conversion of 2-oxoglutarate (alpha-ketoglutarate) to succinyl-CoA and CO(2) during the Krebs cycle	0.016	4.38	0.00	0.0046	1.42E + 07	5.99E-01
NP_001026640.1	427694	SBNO1	15	Chromatin DNA and histone binding protein implicated in the regulation of transcription	0.039	4.02	0.00	0.00054	4.95E + 06	3.03E-01
NP_001026029.1	419257	TPD52L2	20	Member of the tumor protein D52-like family that regulated cell proliferation and metabolic processes	0.00012	5.85	0.00	0.00029	6.16E + 06	0.00E + 00
NP_001004402.1	422842	WDR1	4	Actin binding protein that induce disassembly of actin filaments in conjunction with ADF/cofilin family proteins. Involved in cytokinesis	0.00096	2.91	0.00	0.0025	9.72E + 06	2.96E-01
BAA24137.1	107050620	LOC107050620	36	Chicken high molecular mass nuclear antigen, involved in mRNA translation	0.0005	29.66	0.61	0.0041	8.15E + 06	9.26E + 05
NP_990427.2	395984	HDLBP	9	High density lipoprotein binding protein that plays a role in cell sterol metabolism protecting cells from over-accumulation of cholesterol	0.004	9.27	0.51	0.0074	4.93E + 06	8.55E-01
XP_424271.3	426644	ERMP1	Z	Zinc-binding protease belonging to the peptidase M28 family. Its expression is required in the ovary for the organization of somatic cells and oocytes into discrete follicular structures	0.0001	82.91	5.05	0.018	5.28E + 07	7.65E + 06
XP_021273023.1	110408544	UBR4	20	E3 ubiquitin-protein ligase that is a cytoskeletal component in the cytoplasm and part of the chromatin scaffold in the nucleus	0.00026	11.34	0.84	0.00044	5.09E + 06	8.55E-01
NP_990842.1	396517	SLC2A14	1	Highly conserved integral membrane protein that transport hexoses such as glucose and fructose into cells	0.0004	16.11	1.19	0.04	4.97E + 07	2.16E + 06
XP_015149397.1	416254	GEMIN5	13	Component of the survival of motor neurons (SMN) complex. The SMN complex plays a critical role in mRNA splicing through the assembly of spliceosomal small nuclear ribonucleoproteins (snRNPs), and may also mediate the assembly and transport of other classes of ribonucleoproteins	0.00089	11.12	1.02	0.029	9.70E + 06	1.68E + 06
XP_015128188.1	103906336;104639878;1044883201	STOML2;LOC104014593;LOC101880232	NA	Mitochondrial protein that probably regulates the biogenesis and the activity of mitochondria	0.019	11.40	1.50	0.0076	2.75E + 07	5.51E + 06
NP_989530.1	374025	SMN	Z	Plays a catalyst role in the assembly of small nuclear ribonucleoproteins (snRNPs), the building blocks of the spliceosome. Thereby, plays an important role in the splicing of cellular pre-mRNAs	0.00033	12.39	1.80	0.03	2.09E + 07	2.03E + 06
XP_418564.2	420462	PITRM1	2	Metalloendopeptidase of the mitochondrial matrix that functions in peptide cleavage and degradation rather than in protein processing	0.0049	2.19	0.33	0.0082	7.93E + 06	5.99E-01
P02789.2	396241	TF	9	Iron binding transport proteins responsible for the transport of iron from sites of absorption and heme degradation to those of storage and utilization. Has a role in stimulating cell proliferation	< 0.00010	0.00	458.31	0.00022	1.20E + 00	5.81E + 07
BAU79380.1	395882	BPIFB2	20	Member of the lipid transfer/lipopolysaccharide binding protein (LT/LBP) gene family	< 0.00010	0.00	80.57	< 0.00010	0.00E + 00	4.41E + 07
NP_001263315.1	420898	OVALX	2	Heparin-binding ov-serpin exhibiting antimicrobial activities	0.00057	0	24.69	0.049	0.00E + 00	6.75E + 07
P10184.2	416235	SPIK5	13	Serine protease inhibitor, probably important for the anti-inflammatory and/or antimicrobial protection of mucous epithelia	< 0.00010	0	43.50	< 0.00010	0.00E + 00	1.87E + 07
P67942.1	416236	SPIK7	13	Serine-type endopeptidase inhibitor	< 0.00010	0	27.92	0.014	0.00E + 00	3.04E + 07
CAA55385.1	396151	OVST	1	Serine-type endopeptidase inhibitor	0.0032	0	8.85	0.00055	0.00E + 00	1.44E + 07
NP_001026578.1	426937	PSMA5	26	Component of the 20S core proteasome complex involved in the proteolytic degradation of most intracellular proteins	0.00049	0	2.44	< 0.00010	1.20E + 00	3.68E + 07
NP_989992.1	395381	LOC395381	5	Ovomucin, the glycoprotein responsible for the gel properties of egg white	0.00023	0	14.02	0.0076	0.00E + 00	1.17E + 07
ABU24464.1	395220	ORM1	17	Functions as transport protein in the blood stream. Appears to function in modulating the activity of the immune system during the acute-phase reaction	0.006	0	16.35	0.031	0.00E + 00	2.75E + 07
XP_021263172.1	415523	SNRPA1	10	Involved in pre-mRNA splicing as component of the spliceosome	0.0096	0	3.63	< 0.00010	1.20E + 00	8.84E + 06
XP_009233304.1	395232	EIF4A2	9	ATP-dependent RNA helicase which is a subunit of the eIF4F complex involved in cap recognition and is required for mRNA binding to ribosome	0.0093	0	5.09	0.0028	1.20E + 00	8.17E + 06
XP_015708132.1	419058	PPME1	1	Protein phosphatase methylesterase localized to the nucleus. The encoded protein acts on the protein phosphatase-2A catalytic subunit and supports the ERK pathway through dephosphorylation of regulatory proteins	0.032	0	3.95	0.00029	0.00E + 00	1.76E + 07
NP_990025.1	395430	CDC37	30	Molecular chaperone with specific function in cell signal transduction, binds to numerous kinases and promotes their interaction with the Hsp90 complex	0.026	0	1.25	0.028	8.27E-01	4.63E + 06
NP_001026411.1	423947	ACADSB	6	Short and branched chain specific acyl-CoA dehydrogenase that catalyzes the removal of one hydrogen from C-2 and C-3 of the fatty acyl-CoA thioester, resulting in the formation of trans-2-enoyl-CoA	0.028	0	2.71	0.0012	4.31E-01	6.19E + 06
NP_001007840.1	416955	YWHAH	15	Adapter protein implicated in the regulation of a large spectrum of both general and specialized signaling pathways	0.022	0.02	2.16	0.025	2.02E + 06	1.94E + 07
BAM13279.1	420897	OVALY	2	Serine-type endopeptidase inhibitor	< 0.00010	1.83	95.43	0.016	8.35E + 06	8.44E + 07
XP_014135657.1	422354	RAB39B	4	Small GTPase Rab involved in autophagy and key regulators of intracellular membrane trafficking, from the formation of transport vesicles to their fusion with membranes	0.041	0.03	0.93	0.026	1.39E + 07	7.43E + 07
Q7LZQ2.1	NA	FG	NA	Protein with bacteriolytic function, also associated with the monocyte-macrophage system and immunomodulation	0.0024	0.37	10.39	0.0022	4.31E-01	4.28E + 07
XP_010117667.1	396228	SERPINH1	1	Serine proteinase inhibitor that functions as a chaperone in the biosynthetic pathway of collagen	0.035	0.35	9.93	0.001	1.20E + 00	2.38E + 07
NP_035318.1	417716	PSMC2	1	Component of the 26S proteasome, a multiprotein complex involved in the ATP-dependent degradation of ubiquitinated proteins	0.0018	0.74	5.25	0.00015	1.20E + 00	2.18E + 07
XP_015726668.1	107317943	DTYMK	9	Protein with thymidylate kinase activity that catalyzes the conversion of dTMP to dTDP	0.008	1.12	7.92	0.00012	7.66E-01	4.23E + 06

**Figure 1:**
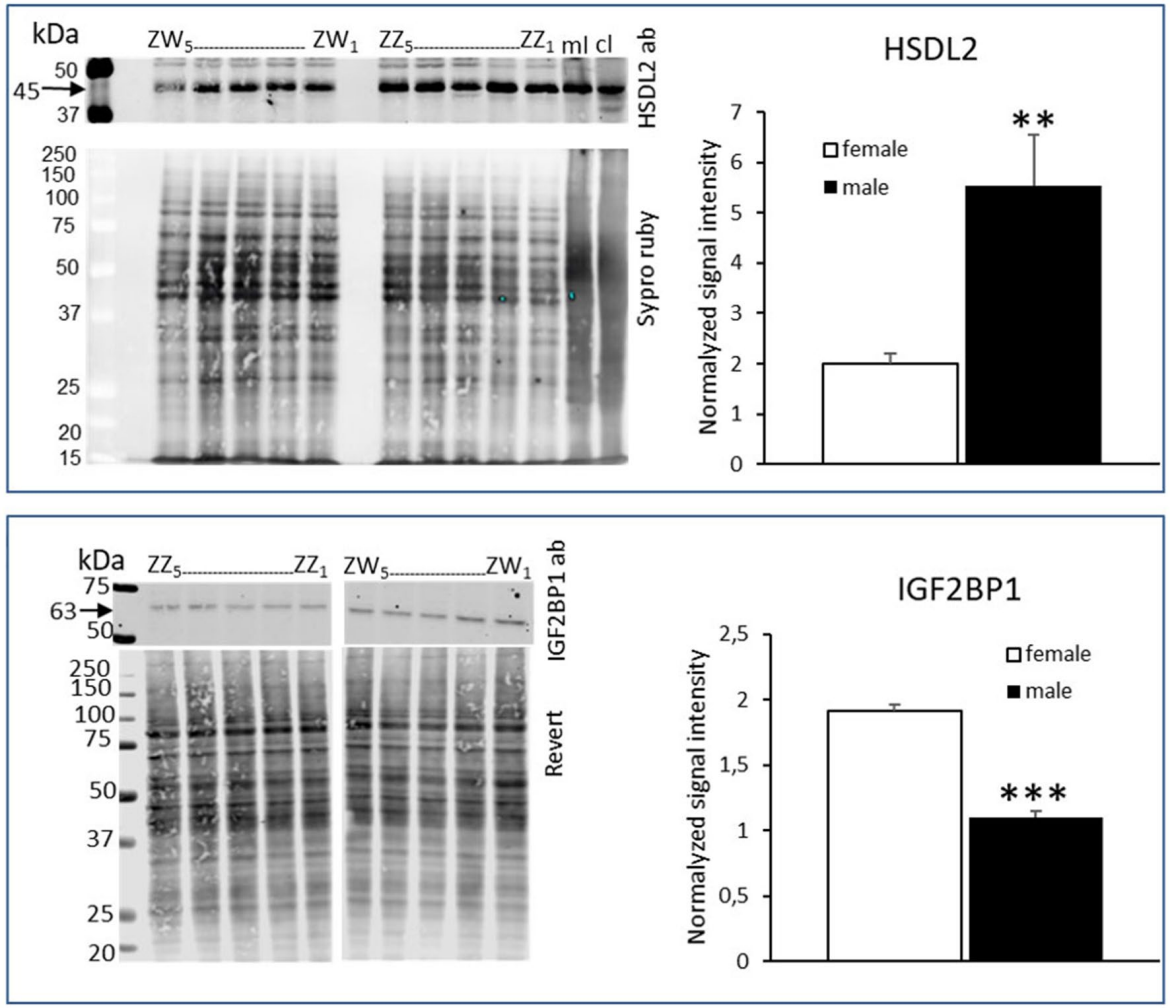
Western blot analysis of HSDL2 (at the top) and IGF2BP1(at the bottom) in chicken male and female PGCs derived in vitro. Western blot analysis was performed on the protein extracts from 5 individual female (ZW_1–5_) and male (ZZ_1–5_) PGCs cultures as described in the Sect. Reverse transcription quantitative real time polymerase chain reaction (RT-QPCR). The pictures of nitrocellulose membrane with blotted proteins after total protein staining and after incubation with HSDL2 and IGF2BP1 are represented. For IGF2BP1 two presented parts of the blot correspond to the same blot. The full-length blots for HSDL2 and IGF2BP1 are presented on the Supplementary Fig. S2. The bands of 45 kDa and of 63 kDa corresponding to HSDL2 and IGF2BP1 proteins respectively are indicated with arrows. Mouse liver (ml); chicken liver (cl) present positive controls. The results on the graphs represent the means ± SEM, *p* < 0.001 (***), *p* < 0.02 (*).

Differential protein abundance found by quantitative proteomic analysis was confirmed by western blot analysis for two proteins, HSDL2 and IGF2BP1, carried out on individual male and female PGCs cultures (Fig. 1, Supplementary Fig. S2), and for ovalbumin, on the pool of male and female individual PGCs cultures (supplementary Fig. S3).

### The male PGC proteome is enriched in proteins encoded in the Z chromosome

We investigated the chromosomal position of the genes encoding differentially abundant proteins and we observed a weak enrichment of genes encoding female overabundant proteins on chromosomes 7 and 25, whereas a strong enrichment of genes encoding male overabundant proteins on chromosome Z (Supplementary Table S3a). Indeed, 15.5% (17 out of 111 unique chicken ID found for proteins more abundant in male PGCs) of more abundant proteins in male PGCs were encoded by genes located on the Z chromosome, whereas only 0.9% (1 out of 108 unique chicken IDs found for proteins more abundant in female PGCs) of female more abundant proteins was linked to the Z chromosome (Table 2, Supplementary Table S3b). When considering all of the identified proteins, only 0.7% (6 out of 764 unique chicken IDs found for all identified proteins) of Z-linked proteins did not change in abundance according to sex. We did not identify any proteins encoded by W-linked genes among proteins more abundant in female PGCs. This was, however, not surprising, considering that the W chromosome is very small and poor in protein-coding genes.

**Table 2:** List of sex chromosome linked genes encoding proteins identified in cultured in vitro chicken PGCs.

NCBI gene name	NCBI gene ID	Sex of preferential abundance	Chromosome
TXNL1	426854	Male	Z
TXN	396437	Male	Z
TTC37	427114	Male	Z
TLN1	395194	Male	Z
TBCA	416367	Male	Z
TARS	427427	Male	Z
SMN	374025	Male	Z
PTGR1	427337	Male	Z
NUP155	427443	Male	Z
IQGAP2	427211	Male	Z
HSDL2	100858057	Male	Z
HINT2	395424	Male	Z
ERMP1	426644	Male	Z
DDX4	395447	Male	Z
AD012Z	407092	Male	Z
ACO1	373916	Male	Z
ACAA2	426847	Male	Z
RPL17	426845	Female	Z
RPS6	396148	none	Z
RPS23	427323	none	Z
LOC430766/LOC107049323	430766/ 107049323	none	W/Z
ISOC1	415601	none	Z
HNRNPK/HNRNPKL	427458 / 426516	none	Z/W
ELAVL2	770158	none	Z
CLTA	427284	none	Z
ATP5I	769146	none	Z
ATP5A1Z/ATP5A1W	374159/431564	none	Z /W

### Overall transcriptome differences do not explain proteome differences found in male and female PGCs

The male to female abundance ratios were higher than two-fold for 68% of the differentially expressed proteins encoded by Z-linked genes. For several of these proteins the ratio was much higher than two, suggesting a positive regulation in addition to a double dose effect.

**Figure 2:**
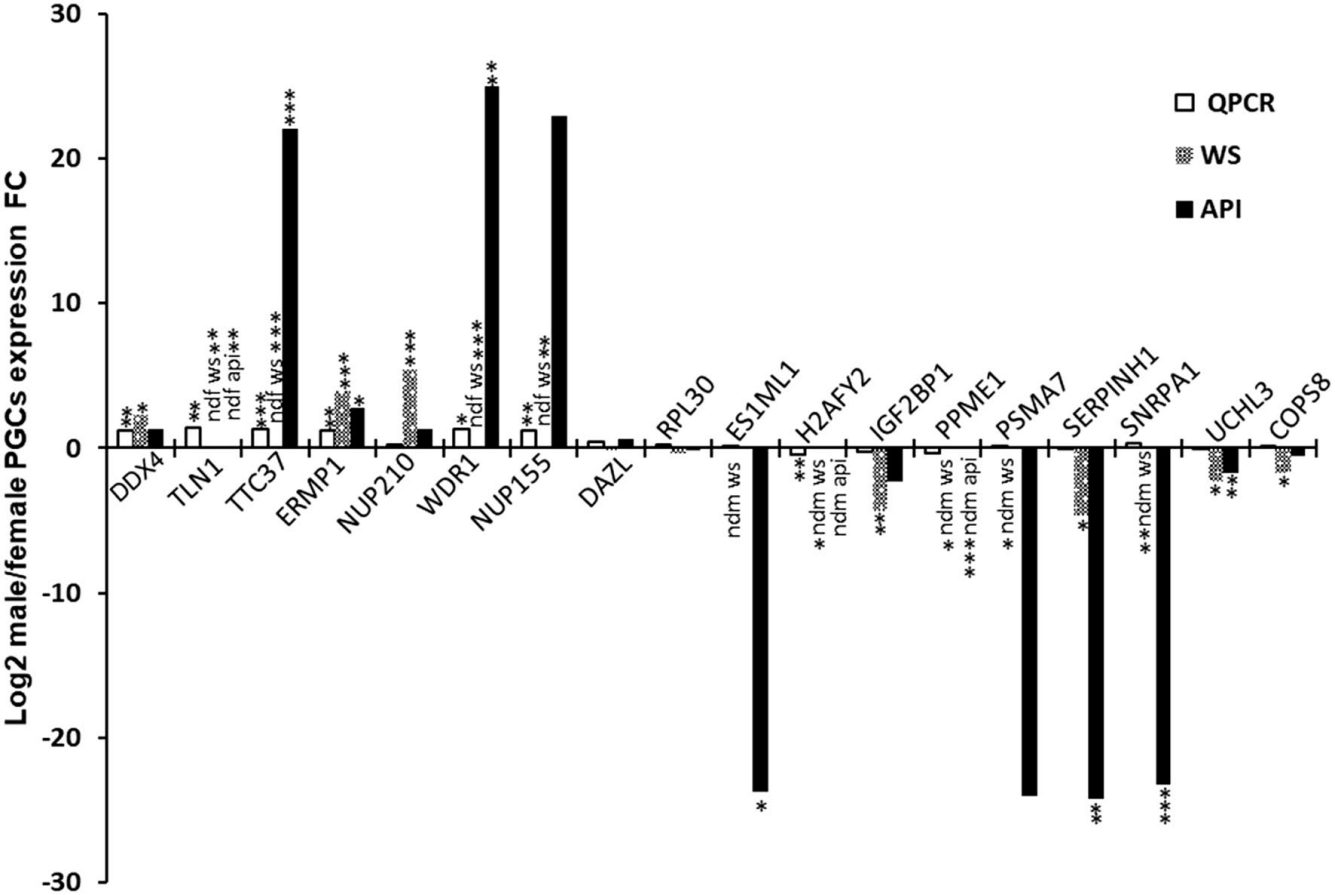
Transcript and protein male/female expression fold change (FC) in cultured in vitro chicken PGCs. Transcript and protein abundances were evaluated by RTQPCR and two label-free proteomic quantitative methods (Spectral Counting and Average Precursor Intensity) respectively, as described in the Sect. Label-free protein quantification. Weighted spectra (WS); Average Precursor Intensity (API); *p* < 0.05 (*); *p* < 0.01 (**); *p* < 0.001 (***); not determined in female PGCs (ndf); not determined in male PGCs (ndm).

We next compared the transcript and protein expression ratios for 5 selected proteins encoded by Z-linked genes and 13 selected proteins encodes by autosomal genes (Fig. 2**)**. The autosomal genes *DAZL* and *Ribosomal Protein L30* (*RPL30*; a germ cell marker and a housekeeping gene, respectively) were chosen as controls, and, as expected, no changes were observed neither at the protein level nor at the transcript level. We observed that for proteins more abundant in male PGCs, transcripts gene expression levels were also significantly higher (6 genes), the single exception being the nucleoporin 210 (NUP210). In contrast, no differences in transcript expression were observed in genes corresponding to female overabundant proteins (9 genes), except for the core histone macro-H2A (H2AFY). For Z-linked genes analysed (*DDX4*, *Tallin-1*; *TLN1*, *Tetratricopeptide Repeat Domain 37; TTC37, Endoplasmic Reticulum Metallopeptidase 1; ERMP1 and Nucleoporin 155; NUP155*), transcript level differences appeared to be due to the previously mentioned lack of Z chromosome dosage compensation in ZZ PGCs, and not due to gene expression upregulation. For these Z chromosome genes and for the autosomal gene WDR1, the increased transcript levels in male PGCs does not appear to be the only mechanism responsible for increased protein accumulation since their protein male/female ratio was much higher than that of the transcript ratios, with the exception of DDX4 (Fig. 2). Instead, post-transcriptional and post-translational mechanisms are likely to be activated to create the higher abundance of these proteins, and this can also explain the high protein male/female ratio for NUP210, whose transcript was not differentially expressed in male and female PGCs. In agreement with this observation, a previous study that compared gene expression and protein abundance in male and female adult chicken tissues described that 30% of all proteins encoded from Z-linked genes showed a significant change in the male/female ratio compared with the corresponding ratio at the RNA level^31,32^.

**Figure 3:**
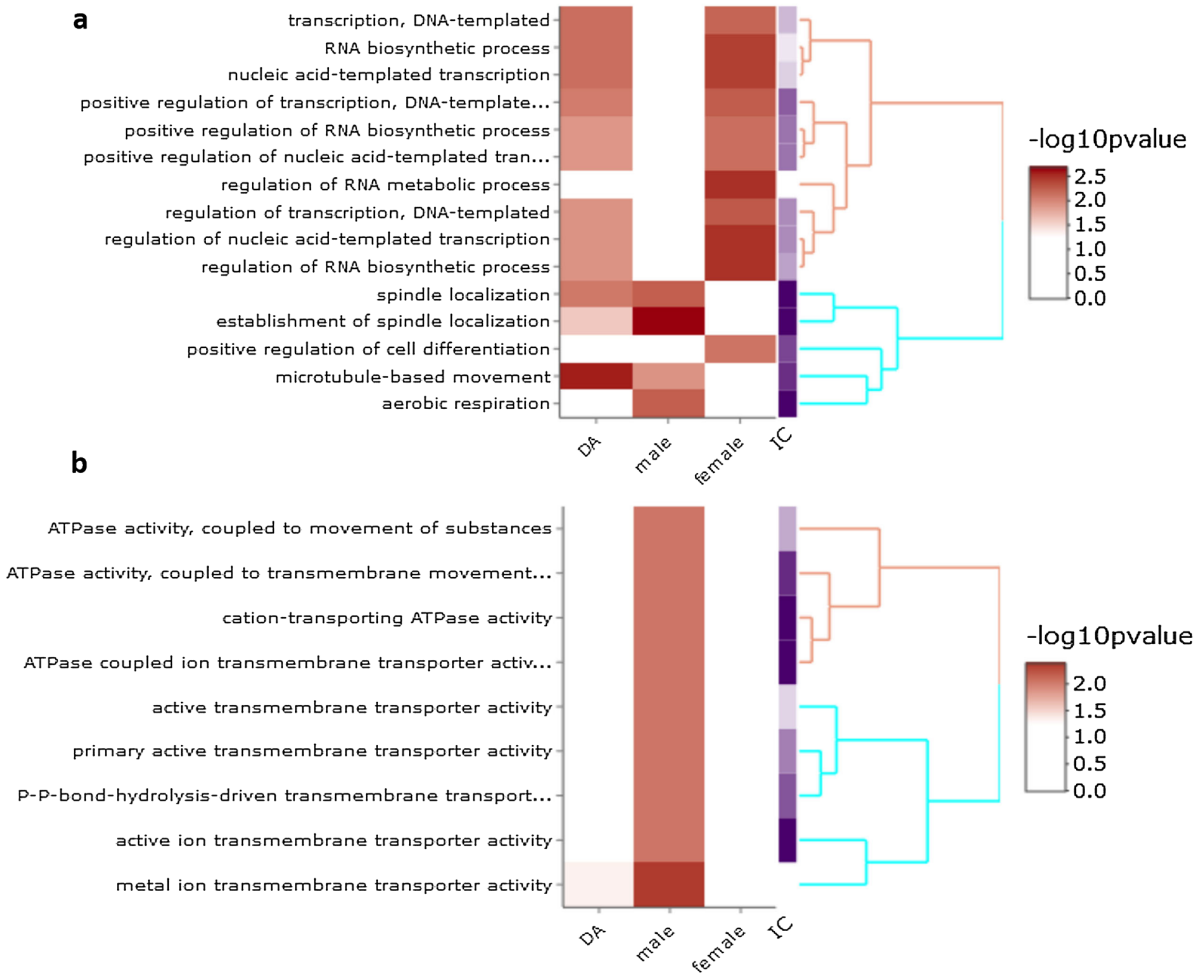
Wang GO terms distance clustering heat map plots for significantly enriched GO terms in differentially abundant proteins in male and female cultured PGCs. DA, GO terms enriched in differentially abundant proteins using as background all identified proteins except components of culture medium specified in the Sect. Female PGCs accumulate proteins from the culture medium and that are not expressed in PGCs; male, GO terms significantly enriched in proteins preferentially abundant in male PGCs; female, GO terms significantly enriched in proteins preferentially abundant in female PGCs; IC, informational content. Functional analysis and its visualisation were performed using ViSEAGO^24^. (**a**), Go terms Biological Process (BP) clustering heatmap plots; (**b**), Go terms Molecular Function (MF) clustering heatmap plots.

The differential expression of both autosomal and Z-linked genes in male PGCs in the absence of surrounding gonadal somatic cells or sexual hormones suggests that their molecular sex differentiation is cell autonomous, as previously suggested in studies performed in murine PGCs and chicken embryonic tissues^32–34^. The substantial sexual differences in the protein abundances in pregonadal PGCs observed in this study may be the consequence of the cumulative effect of uncompensated Z-linked gene products. This observation may be linked to the proposed role of uncompensated dosage of Z-linked genes in the molecular sexual identity of early chicken embryo^32^.

### Female PGCs accumulate proteins from the culture medium

Several of the proteins that were highly overabundant in female PGCs (Table 1, supplementary Table S2c), transferrin, (TF), BPIFB2, SPINK5, SPINK7, ovostatin (OVST), ovomucin, alpha subunit (LOC395381, OVOA), orosomucoid 1 (ovoglycoprotein) ORM1, ovalbumin-related protein Y (OVALY, SERPINB14B), ovalbumin-related protein X (OVALX (SERPINB14C), VMO1, ovalbumin (OVAL, SERPINB14), were not expected to be present in PGCs, as they are oviduct- or serum-specific proteins. Using RT-qPCR analysis, we observed that female PGCs have no or very low mRNA expression for these proteins (Supplementary Fig. S4). We, thus, suspected that female PGCs were able to selectively pinocytosis and/or transport these proteins from the extracellular culture medium. To confirm this hypothesis, we analysed two commercial protein products added to cell culture medium: ovalbumin (albumin from hen egg white; SIGMA) and chicken serum (SIGMA) by nanoLC-MS/MS analysis (Supplementary Methods). All the proteins detected in female PGCs for which we could not find corresponding mRNA expression were identified by this analysis, with the exception of vitelline membrane outer layer 1 homolog (VMO1) and ORM1 (Table 3, Supplementary Table S4). Among these proteins BPIFB2 (also known as protein TENP, G2), OVOA (also known as LOC395381), SPINK5, SPINK7, OVAL (also known as SERPINB14), OVALX, OVALY, OVST were identified in the commercial ovalbumin product while TF was found in both albumin from hen egg white and chicken serum. VMO1, another component of the egg white, was not identified in the ovalbumin fraction used in PGCs culture medium, probably because of its lower abundancy in egg white compared with the rest of the proteins which may have prevented its detection^35^. In view of the fact that these components were also present in the male PGC culture medium, these results suggest that female PGCs are able to differentially import proteins from the medium compared with male PGCs, indicating an intrinsic capacity of female germ cells to transport and accumulate proteins.

**Table 3:** Proteins overabundant in female PGCs and identified by LC–MS/MS Analysis of chicken ovalbumin and chicken serum.

Albumin from hen egg white (SIGMA)						
Group ID	Subgroup ID	subgroup protein ID	UniProt Protein ID	Description	Gene Name	emPAI
a1	a1.a1	a1.a1.a1	A0A2H4Y814	CHICK OVA	OVA	7.6E + 08
	35 other proteins for group a1			CHICK OVA	OVA	5.4E + 08**
a2	a2.a1	a2.a1.a1	P01014	CHICK Ovalbumin-related protein Y	OVALY	153
	a2.a2	a2.a2.a1	A0A1D5P531	CHICK Uncharacterized protein	OVALX	3.6
	a2.a2	a2.a2.a2	A0A1D5PI58	CHICK Uncharacterized protein	OVALX	3.2
a3	a3.a1	a3.a1.a1	A0A1D5P4L7	CHICK Ovotransferrin	N/A	2
a3	a3.a2	a3.a2.a1	P02789	CHICK Ovotransferrin	TF	1.5
a4	a4.a1	a4.a1.a1	P01005	CHICK Ovomucoid	N/A	55
a4	a4.a1	a4.a1.a2	A0A1D5NYW	CHICK Ovomucoid	SPINK7	35
a4	a4.a1	a4.a1.a3	B6V1G0	CHICK Ovomucoid	N/A	35
a5	a5.a1	a5.a1.a1	Q98UI9	CHICK Mucin-5B	MUC5B	0.2
a5	a5.a1	a5.a1.a2	A0A1D5PPR9	CHICK Uncharacterized protein	LOC395381	0.2
a6	a5.a1	a5.a1.a3	F1NZY2	CHICK Uncharacterized protein	LOC395382	0.2
a5	a6.a1	a6.a1.a1	P20740	CHICK Ovostatin	N/A	0.3
a6	a6.a1	a6.a1.a2	A0A1D5NT83	CHICK Uncharacterized protein	OVST	0.3
a6	a6.a1	a6.a1.a3	A0A1D5P3R8	CHICK Uncharacterized protein	OVST	0.3
a6	a6.a1	a6.a1.a4	A0A1L1RUH2	CHICK Uncharacterized protein	OVST	0.3
a9	a9.a1	a9.a1.a1	A0A146J2W9	CHICK Protein TENP	TENP	0.4
a9	a9.a1	a9.a1.a2	A0A146J2X3	CHICK Protein TENP	TENP	0.4
a9	a9.a1	a9.a1.a3	A0A146J2Y8	CHICK Protein TENP	TENP	0.4
a9	a9.a1	a9.a1.a4	A0A146J2Z8	CHICK Protein TENP	TENP	0.4
a9	a9.a1	a9.a1.a5	A0A146J302	CHICK Protein TENP	TENP	0.4
a9	a9.a1	a9.a1.a6	A0A146J377	CHICK Protein TENP	TENP	0.4
a9	a9.a1	a9.a1.a7	A0A146J3I5	CHICK Protein TENP	TENP	0.4
a9	a9.a1	a9.a1.a8	A0A1L1RMA4	CHICK Protein TENP	BPIFB2	0.4
a9	a9.a1	a9.a1.a9	I0J173	CHICK OvoglobulinG2	G2	0.4
a9	a9.a1	a9.a1.b10	I0J174	CHICK OvoglobulinG3	G2	0.4
**chicken serum (SIGMA)**						
a3	a3.a1	a3.a1.a1	A0A1D5P4L7	CHICK Ovotransferrin	TF	182.3
a3	a3.a2	a3.a2.a1	Q4ADJ6	CHICK Ovotransferrin	TFEW	111.9
b17	b17.a1	b17.a1.a1	P10184	CHICK Ovoinhibitor OS	OIH	9.0
b17	b17.a2	b17.a2.a1	F1NMN2	CHICK Uncharacterized protein	SPINK5	9.0

**Mean emPAI for 35 proteins from group a1 identified as Ovalbumin.

### Post-transcriptional RNA and post-translational protein processing are enhanced in female chicken PGCs

**Figure 4:**
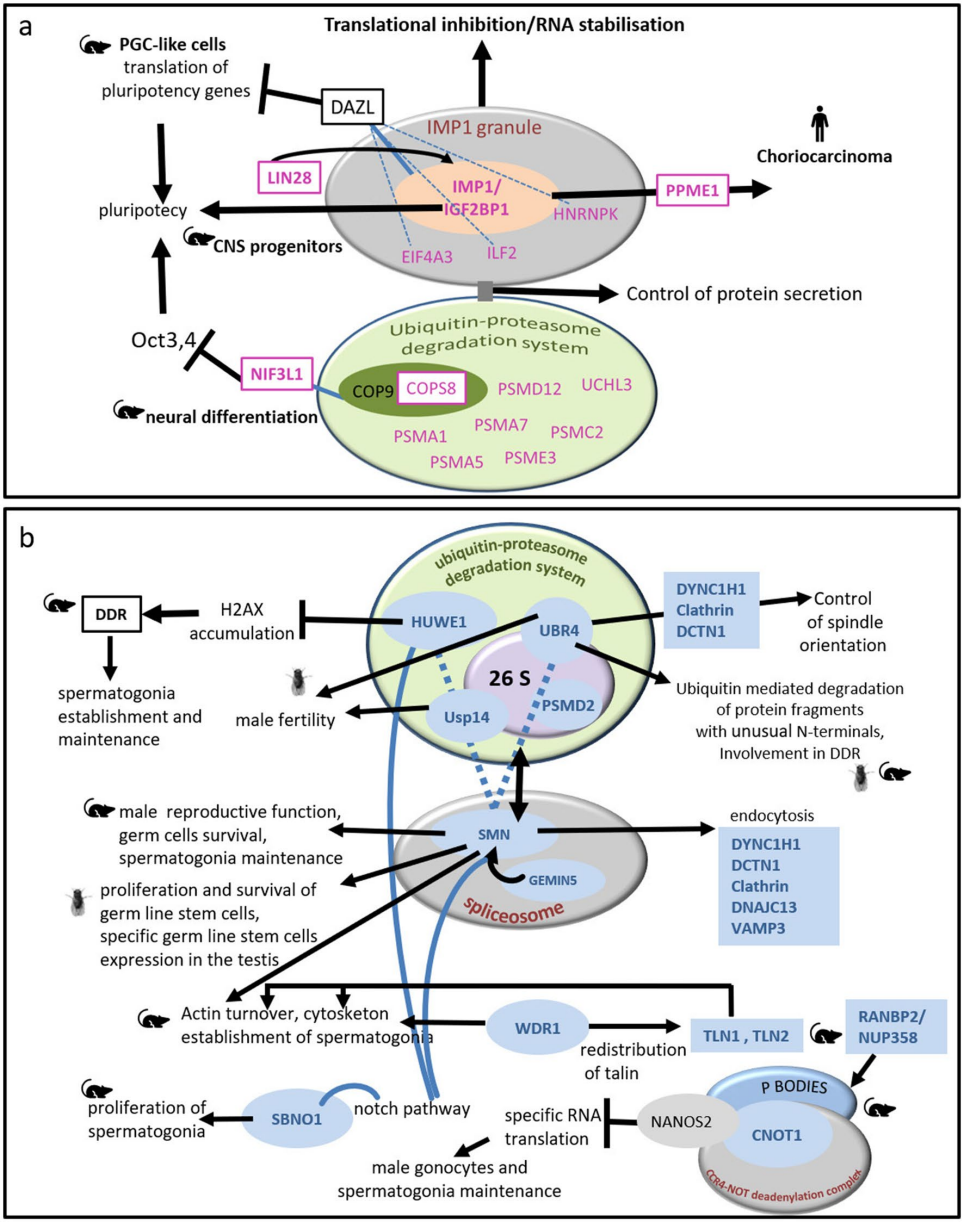
Crosstalk of pathways involving orthologs of chicken proteins preferentially abundant in chicken female (**a**) and male (**b**) PGCs based on the literature (for references see Sects. 3.6 and 3.7). Proteins in pink and blue correspond to the orthologs of proteins identified as overabundant in chicken female and male PGCs respectively. Pointed and blunted arrow heads represent activation and inhibition respectively.

We carried out a functional pathway analysis on proteins found in male and female PGCs. This functional analysis suggested increased transcriptional and translational processing in female PGCs compared to male PGCs, indicated by the significant overrepresentation of the “Biological Process” (BP) GO terms “RNA transcription and its regulation”, “RNA biosynthetic processes”, “regulation of RNA biosynthesis” and “regulation of RNA metabolism” (Fig. 3a; Supplementary Table S5). Based on the functional analysis and literature searches on overabundant proteins in female PGCs, we elaborate on how these proteins are interrelated, as summarized in Fig. 4a and as detailed below.

#### Germ cell phenotype of female PGCs is promoted by an enrichment in proteins related to RNA metabolism and protein processing

In germ cells (and somatic cells in a lesser extent) cytoplasmic untranslated mRNAs accumulate in ribonucleoprotein (RNP) complexes, which are important posttranscriptional regulators of gene expression during development with functions in mRNA degradation, storage, sorting, and transport. A subset of more abundant female PGCs proteins has been found to be components of RNP granules in murine PGCs-like cells including: insulin growth factor 2 binding protein 1 (IGF2BP1, also known as IMP1), Interleukin Enhancer Binding Factor 2 (ILF2), Eukaryotic Translation Initiation Factor 4A3 (EIF4A3) and Heterogeneous Nuclear Ribonucleoprotein K (HNRNPK)^36^. In these cells, IGF2BP1 is associated with the germ cell marker DAZL in RNP granules, whose role is to carry out translational repression of differentiation, pluripotency and apoptosis related RNAs^36,37^. Interestingly, female PGCs were enriched in proteins involved in secretion and the ubiquitin–proteasome degradation system compared to male PGCs (RABB1, RAB11B, UBE2V2, UCHL3, PSMA1, PSMA5, PSMA7, PSME3, PSMC2, PSMD12, COPS8). The respective mRNAs of these proteins are known to be components of IGF2BP1-containing RNP granules^37^. These results suggest that in female PGCs, there is a preponderant cooperation between RNP granules, the protein-processing organelle machinery and the ubiquitin–proteasome degradation system to govern RNA metabolism, protein synthesis, processing, and degradation.

Another protein related to the promotion of ‘germ cell-ness’ mediated by IGF2BP1 signalling, the post-transcriptional regulator LIN-28 homolog A (LIN28A), was overabundant in female PGCs. The expression of LIN28A is restricted to PGCs and premeiotic germ cells in foetal mouse and human ovaries^38,39^, where it is known to promote pluripotency and to be involved in PGC formation. LIN28A has been shown to interact mechanistically and functionally with IGF2BP1 to regulate the insulin-like growth factor 2-mammalian target of rapamycin (IGF2-mTOR) signalling in neural progenitor cells, a pathway that is also essential for chicken PGCs survival and proliferation^5,40^. Moreover, LIN28A promotes *IGF2BP1* expression, allowing post-transcriptional repression of differentiation genes in foetal mouse neural stem cells^41^. Both proteins are also indirectly related through the regulation of pluripotency gene SOX2 in neural precursor cells and in murine in vitro derived PGC-like cells^36,42^. Accordingly, LIN28A and IGF2BP1 could cooperate in chicken PGCs in maintaining the fine balance between germ cell-ness and pluripotency and this mechanism could be enhanced in chicken female PGCs. Sex differences observed in LIN28A protein expression may indicate differences in the pluripotency state of male and female PGCs.

In addition to the possible role of IGF2BP1 in the regulation of pluripotency in chicken PGCs IGF2BP1 may also promote female PGCs migration and gonad colonisation through protein phosphatase methyl esterase 1 (PPME1) overabundant in female PGCs, as PPME1 has been found to be novel effector of IGF2BP1 in promoting migration and invasion of human choriocarcinoma cells^43^.

#### Female PGCs may regulate the activity of the ubiquitin–proteasome system through the activity of COP9 signalosome

The COP9 signalosome (CSN) is a highly conserved protein complex associated with de-ubiquitination activity and protein kinase activities capable of phosphorylating important signalling regulators. NGG1 Interacting Factor 3 Like 1 (NIF3L1) and COP9 Signalosome Subunit 8 (COPS8/CSN8) were overabundant in female PGCs. NIF3L1 is a transcriptional corepressor through its interaction with CSN2/COPS2 (a subunit of the COP9 signalosome complex or CSN), negatively regulating the expression of OKT-3/4, another master regulator of pluripotency and partner of Sox2, and promoting neural differentiation^36,44^. Female PGCs might have an enhanced CSN activity in order to control ubiquitin–proteasome-mediated protein degradation to regulate their state of differentiation.

#### Female PGCs control transcription through an enrichment in nucleosome proteins

Several nucleosome proteins involved in nuclear architecture, chromatin state and epigenetic regulation of transcription were overabundant in female PGCs. Among them, three subtypes of histone H1 (H1.10 Linker Histone; H1FX, Histone H1.03; HIST1H103, histone cluster 1, H1.11R) and the core histone macroH2A2 (MACROH2A2). Although a higher abundance of H1 linker histone subtypes in female PGCs in comparison to male PGCs after gonad differentiation have been described in the mouse^45^, ours is the first report of sex-related H1 differences in germ cells before gonad differentiation. These results indicate that sexually dimorphic nucleosome regulation of transcription is present in chicken PGCs, before PGC differentiation begins.

### Male chicken PGCs proteome suggests common features with spermatogonia

Functional analysis of differentially abundant proteins in male PGCs pointed to cytoskeleton organisation differences and increased metabolism, as suggested by the enriched BP GO terms “spindle localisation” and “aerobic respiration”, as well as with the MF GO terms “ATP-ase activity coupled to substances”, “transmembrane movement” and “transmembrane transport” (Fig. 3b). The crosstalk of pathways in which these proteins are involved and which may operate in chicken ZZ PGCs based on the literature is summarized in Fig. 4b.

These GO terms reflect the enrichment of the male chicken PGC proteome in proteins known to play an essential role in the post-natal establishment and maintenance of spermatogonia in several species. PGCs, as opposed to gonocytes (quiescent gonadal embryonic germ cells), share some properties with spermatogonia, in particular the ability to proliferate and migrate towards the somatic niche. Our results suggest that these proteins are necessary in pre-gonadal male PGCs for these processes and that their role may, therefore, be in part similar to that in spermatogonia maintenance.

#### The germ cell phenotype of male PGCs is promoted by an enrichment in proteins related to the ubiquitin–proteasome protein degradation system

Several proteins associated with the Ubiquitin-26S proteasome complex were preferentially abundant in male PGCs, namely Ubiquitin Protein Ligase E3 Component N-Recognin 4 (UBR4), WWE Domain Containing E3 Ubiquitin Protein Ligase 1 (HUWE1), non-ATPase regulatory subunit 2 (PSMD2), 26S proteasome co-factor thioredoxin-like protein 1 (TXNL1)^46,47^, and a chicken orthologue of Ubiquitin Specific Peptidase 14 (USP14)^48^. A number of these proteins are known to specifically direct protein degradation in male spermatogonia or sperm cells. HUWE1, known to ubiquitinate core histones, has been recently reported to be essential to the spermatogonia establishment and maintenance by preventing genomic instability via suppressing the DNA Damage Response (DDR) through blocking the accumulation of Histone H2AX in mice^49^. Moreover, UBR4, an E-3 ligase having “N-end rule” specificity has been shown to be involved in the degradation of histone-binding complex HAT1/RBBP4/RBBP7 in Drosophila and mouse myofibers^50^.The proteins in this complex are known to be involved in DNA repair^51–53^. According to these results, male PGCs could promote the ubiquitin–proteasome system to control core histone half-life. Additionally, UBR4 and USP14 are essential for male but not for female fertility in several species^54–56^.

#### Spinal motor neuron (SMN) is a central functional node in the differential male PGC proteome

The protein, survival motor neuron (SMN), as well as its partner and translational activator, GEMIN5^57,58^, were significantly more abundant in male PGCs. Using literature mining, SMN appears to be a central functional node in the network of the overabundant proteins in male PGCs. SMN protein seems to interrelate different processes essential for male fertility involving intracellular trafficking, protein degradation and translational silencing^57^, and to directly interact with several of its components. SMN is able to interact with the ubiquitin–proteasome-degradation system to control protein degradation including E3-ligases^59–61^. In our dataset, this relationship is suggested by the co-accumulation of SMN with the E3 ligases HUWE1 and UBR4 in male PGCs.

SMN is present in the cell nucleus, localized to subnuclear bodies near coiled bodies containing high concentrations of small ribonucleoproteins (snRNPs), and is involved in the biogenesis of snRNPs, the building blocks of the spliceosome^62^. One of snRNPs components, small nuclear ribonucleoprotein polypeptide F (SNRPF), was also overabundant in male PGCs. SMN is essential for male but not female reproductive function and is involved in the germ cell development and survival and spermatogonia maintenance in the mouse^63,64^. In Drosophila, SMN expression is essential for survival and proliferation of male germline stem cells while it is not expressed in spermatocytes or the somatic niche^65^.

In Drosophila, SMN is implicated in the regulation of germline nuclear organization through the connection of U bodies (structures rich in uridine-rich small nuclear ribonucleoproteins involved in key steps of pre-mRNA processing) and processing bodies (P-bodies; mRNA-degrading RNP granules)^66^. Other two proteins known to be involved in the formation of P-bodies were more abundant in male PGCs, namely E3 SUMO-protein ligase RANBP2 (also known as NUP358)^67,68^ and CCR4-NOT transcription complex subunit 1(CNOT1). In mice, RANBP2 is known to be essential for male fertility^68^ and CNOT1, interacting with NANOS2 in both male gonocytes and spermatogonial stem cells (SSCs) maintenance, is involved in suppression of specific RNAs promoting male differentiation fate of germ cells and blocking the female program^69^. This suggests that in chicken male PGCs, SMN, CNOT1 and RANBP2 could be connected through their involvement in the function of P bodies and, consequently, in translational silencing.

SMN is a repressor of the cell-fate Notch signalling pathway^70^. The involvement of Notch signalling in male PGCs homeostasis was indicated with the co-accumulation of Strawberry Notch1 (SBNO1), a helicase-related nuclear factor which is known to promote the proliferation of SCCs in mouse neonatal testis^71^. Interestingly, Huwe1 has also been found to regulate Notch signalling in neural progenitors^72^.

Our results indicate that the protein SMN was highly inter-connected with other proteins expressed more abundantly in male PGCs (Fig. 4b), suggesting its central role in male PGCs homeostasis.

#### Intracellular trafficking and cytoskeletal remodelling are enhanced in male PGCs

Pre-gonadal male and female PGCs migrate towards and colonize the gonads during embryonic development. These two fundamental PGCs functions require changes in the actin cytoskeleton as well as enhanced intracellular trafficking. Our results indicate that male PGCs are enriched in proteins related to these processes. Several proteins related to the actin cytoskeleton and cytokinesis were overrepresented in the male PGCs proteome, namely WDR1 (also known as AIP1)^73^, SMN^74,75^, Talin 1 (TLN1) and Talin 2 (TLN2)^76–78^. WDR1 is essential for the migration of germ cells towards the testicular cords in the post-natal mouse testis^73^. WDR1 and talins are known to be functionally interconnected during cytoskeletal re-arrangements. For instance, the redistribution of TLN1 and TLN2 is regulated by WDR1 in a process mediated by integrins during platelet activation in the mouse^79^. Integrin β1 receptors are involved in chicken PGCs migration during embryonic development^80^. Integrin-mediated signaling is mediated by the E3 ubiquitin ligase enzyme UBR4, which was also more abundant in male PGCs together with the protein Clathrin. Both proteins form meshwork structures involved in membrane morphogenesis and cytoskeletal organization^81^. Directly linked with this, the biological process “spindle localization” was enriched in male PGCs according to functional analysis, which involved Dynein Cytoplasmic 1 Heavy Chain 1 (DYNC1H1) and Dynactin Subunit 1 (DCTN1). Indeed, these proteins and UBR4 have been related to equivalent processes in neural progenitors^82,83^.

An enhanced membrane and intracellular trafficking, which is a feature compatible with the pro-migrating phenotype of PGCs, was also suggested in male PGCs by proteomic results. For instance, SMN, HUWE1 and UBR4 are involved in intracellular trafficking and promote endocytosis^84,85^. Moreover, male PGCs were enriched in several endosome components including the proteins Clathrin, Vesicle Associated Membrane Protein 3 (VAMP3), DYNC1H1, DCTN1 and DnaJ Heat Shock Protein Family (Hsp40) Member C13 (DNAJC13/RME8). Moreover, Clathrin-mediated trafficking is a major mechanism for internalization of membrane receptors-ligand complexes^86^ including those of Insulin^87^, fibroblast growth factor (FGF)^88^ and bone morphogenetic proteins (BMPs)^89^, which are components of culture medium essential for survival and proliferation of PGCs^5^. Thus, enhanced trafficking may have a consequence on the sensitivity of PGCs to these molecules and on their signaling as it has been described in other cell models^90–92^.

In conclusion, male and female chicken PGCs propagated in vitro showed early proteome differences in agreement with a cell autonomous sex determination during early embryonic development which is independent of sex hormones. Moreover, our proteomic results show that the absence of a general mechanism of dosage compensation of Z-linked genes in chicken extends to pre-gonadal PGCs. Indeed, this phenomenon had been documented at transcriptional and translational levels in adult chicken tissues, and at a transcriptional level for embryonic tissues, but not in embryonic germ cells. The cumulative effect of the double dosage of proteins encoded by Z-linked genes in male PGCs may be one specific factor governing cell autonomous sex determination of chicken PGCs. In consequence, ZZ and ZW PGCs may develop different molecular strategies to achieve equivalent functions such as the blocking of sex-specific differentiation and repression of opposite sex developmental pathways. Furthermore, specific differences in ZZ and ZW PGCs overabundant protein sets indicate an early programming of male and female germ cell fates. This study provides the first molecular support that PGCs may be subjected to sex specific regulation independent of the in vitro environment. Sex specific adaptation of in vitro culture conditions for chicken PGCs would be useful to improve quality of these cells for the management of avian genetic diversity. Based on our findings, Insulin, FGF and BMP signaling and the pluripotency state of PGCs are potential candidate targets for further investigation.

## Supplementary Information

Supplementary Information 1.

Supplementary Information 2.

Supplementary Information 3.

Supplementary Information 4.

Supplementary Information 5.

Supplementary Information 6.

## Data Availability

The mass spectrometry proteomics data have been deposited to the ProteomeXchange Consortium via the PRIDE89 partner repository with the dataset identifier PXD022452 and 10.6019/PXD022452.
