# Supplementary material for: Protein expression reveals a molecular sexual identity of avian primordial germ cells at pre-gonadal stages
Source: Sci Rep. 2021 Sep 28;11:19236. doi: 10.1038/s41598-021-98454-2 (PMC8478952; doi:10.1038/s41598-021-98454-2)

SUPPLEMENTARY INFORMATION

SUPPLEMENTARY MATERIALS AND METHODS

In-gel Protein Digestion and NanoLC-MS/MS for PGC protein identification

Gel pieces were washed in water, acetonitrile (1:1) for 5 min followed by a second wash in 100% acetonitrile for 10 min. Cysteine reduction and alkylation were performed by successive incubations with 10 mM dithiothreitol in 50 mM NH_4HCO_3 (30 min, at 56°C) and 55 mM iodoacetamide in 50 mM NH_4HCO_3 (20 min, at room temperature, in the dark). Gel pieces were incubated with 50 mM NH_4HCO_3 and acetonitrile (1:1, 10 min) followed by 100% acetonitrile (15 min). Digestion was carried out overnight using 25 mM NH_4HCO_3 with Trypsin (Sequencing grade, Roche diagnostics, Paris, France) at 12.5 ng/µl. Peptides were extracted from the gel by incubation in 5% formic acid and then in 100% acetonitrile. The two peptide extracts were pooled with the initial digestion supernatant and dried using a SPD1010 speedvac system (ThermoSavant, Thermofisher Scientific). For each protein band, the resultant peptide mixture was reconstituted with 30 µl of 0.1% formic acid, 2% acetonitrile, sonicated for 10 min, and analysed by nanoLC-MS/MS in triplicate. All experiments were performed on an LTQ Orbitrap Velos Mass Spectrometer coupled to an Ultimate® 3000 RSLC chromatographer (Thermo Fisher Scientific, Bremen, Germany). Samples were loaded on a trap column (Acclaim PepMap 100 C18, 100 mm i.d. x 2 cm long, 3 mm particles) and desalted for 10 min at 5 µL/min with 4% solvent B. Mobile phases consisted of (A) 98% water/2% acetonitrile in presence of 0.1% formic acid, and (B) 84% acetonitrile 16% water in presence of 0.1% formic acid. Separation was conducted using a nano-column (Acclaim PepMap C18, 75 mm i.d x 50 cm long, 3 mm particles) at 300 nl/min by applying gradient consisted of 4-30% B during 80 min, 30 to 55% B for 10 min, 55 to 99% for 1min, constant 99% B 20 min and return to 4 % B in 1 min. The eluate was nano-electrosprayed through a Thermo Finnigan Nanospray Ion Source 1 with a SilicaTip emitter of 15 µm inner diameter (New Objective, Woburn, MA, USA).

Standard mass spectrometric conditions for all experiments were spray voltage 1.2 kV, no sheath and auxiliary gas flow; heated capillary temperature, 275°C; predictive automatic gain control (AGC) enabled, and an S-lens RF level of 60%. MS and MS/MS spectra were acquired using Xcalibur software (version 2.1; Thermo Fisher Scientific, San Jose, CA). The instrument was operated in positive ion mode using data-dependent acquisition mode in 300–1800 m/z range with a targeted resolution set at 60,000. The 20 most intense ions with charge states ≥2 were sequentially isolated (2 m/z isolation width; 1 micro scan) and fragmented using CID (collision-induced dissociation) mode (normalized collision energy of 35% and wideband-activation enabled). Dynamic exclusion was active during 30 s with a repeat count of 1. Internal calibration was applied using a lock mass with Polydimethylcyclosiloxane (m/z, 445.1200025) ions.

MS/MS ion searches were performed using Mascot search engine v 2.3 (Matrix Science, London, UK) via Proteome Discoverer 2.1 software (ThermoFisher Scientific, Bremen, Germany). MS and MS/MS data were matched automatically against the "chordata" section of a locally maintained copy of nr NCBI (download january 2018, 9 822 753 sequences). The search parameters included trypsin as a protease with two allowed missed cleavages and carbamidomethylcysteine, methionine oxidation and acetylation of N-term protein as variable modifications. The tolerance was set to 5 ppm for precursors and 0.8 Da for fragment ion matches. Mascot results obtained from the target and decoy databases searches were integrated into Scaffold software (v 4.8, Proteome Software, Portland, USA) to validate protein identifications. Peptide and proteins identifications were accepted if they could be established at greater than 95.0% probability as specified by the Peptide Prophet algorithm and by the Protein Prophet algorithm { HYPERLINK "#_ENREF_24" \o "Keller, 2002 #242" }, respectively. Protein identifications were accepted if they contained at least two identified peptides.

LC-MS/MS Analysis of chicken ovalbumin and chicken serum

Ten micrograms of chicken serum (Sigma-Aldrich, France) and of chicken ovalbumin (albumin from hen egg white; Sigma-Aldrich, France) were denatured by reduction in DTT 10 mM in boiling water for 10 min and were then shortly migrated in SDS-PAGE 10% . After migration the gel was stained by coomassie blue and the bands were excised from the the gel and sent to PAPPSO platform facilities (http://pappso.inrae.fr/). Exised gel bands containing samples were washed with 50 µl of formic acid 1°% in ethanol 40%, followed by three 15 min washing by 50 µl acetonitrile 50% in NH4HCO3 50 mM. Cysteine reduction and alkylation were performed by successive incubations with 10 mM dithiothreitol in 50 mM NH4HCO3 (30 min, at 56°C) and 50 mM iodoacetamide in 50 mM NH4HCO3 60 min, at room temperature, in the dark). Gel pieces were then succesively washed in 50µl of 50 mM NH4HCO3/50% acetonitrile and in 50µl acetonitrile 100% . After proteolytic digestion carried out overnight using 100 ng Trypsin (Sequencing grade, Roche diagnostics, Paris, France) in 25 mM NH4HCO3 and stopped by TFA 0.1%, the peptides were successively extracted by acetonitrile 40%/TFA 0.1% and by acetonitrile 100%. The peptide extracts were dried at speed vac and resuspended in 50 µL acetonitrile 2%/TFA 0.1% before to be loaded on the LC-MS/MS system. Sample were injected and preconcentrated on a precolumn (Acclaim PepMap C18 particle 5 µm size, 5 mm length, 300 µm i.d., Thermo Fisher Scientific) at 20 µL/min with 0.08 % TFA in 2 % ACN in 2 min, followed by a separation on reverse phase separating column (Acclaim PepMap RSLC nanoViper, C18 particle 2 µm size, 150 mm length, 75 µm i.d., Thermo Fisher Scientific). The peptides were eluted with a multi-step gradient from 1 to 35 % of ACN with 0,1% formic acid for 79 min at 300 nL/min for a total run of 90 min. MS scans were acquired in a mass range of m/z 300-1400 at a resolution of 15000 in the orbitrap analyser. The 8 most intense ions were selected for CID MS/MS with a normalised collision energy of 35 in the ion trap.

All MS/MS spectra were searched against UniprotKB database Gallus gallus using X!TandemPipeline (version 3.4.3), the open search engine developped by PAPPSO ({ HYPERLINK "http://pappso.inra.fr/bioinfo/xtandempipeline/" }). Precursor mass tolerance was 10 ppm and fragment mass tolerance was 0.5 Da. Data filtering was achieved according to a peptide E-value < 0.01, protein E-value < 10e-4 and to a minimum of two identified peptides per protein.

Western blot

PGCs were centrifuged at 1,000g for 10 min. The cell pellet was washed two times with 50 volumes of Phosphate saline buffer (PBS) without CaCl2 and MgCl2, and pellet was stored at –80°C until use. Cells samples were sonicated in Tris-HCl 50mM PH 8.8 buffer containing Urea 6M, 4% SDS and protease inhibitors (Roche Switzerland). After allowing extracted proteins to solubilize for 20 min at room temperature with continuous shaking, cell homogenates were centrifuged at 12,000g for 30 min at 4 C°, and protein quantification was measured from protein extracts (Thermo Scientific Pierce BCA Protein Assay Kit). For HSDL2 and IGF2BP1 analysis twenty µg of total protein extracts were separated on 10% SDS-PAGE minigel. For ovalbumin analysis 3.3 µg of total protein extracts were separated on 10%, 12% or 15% minigel depending on experience. Separated proteins were blotted onto nitrocellulose membranes and blocked for one h in 5% skimmed milk TBS-Tween 20. The membranes were then incubated with first antibodies in 5% skimmed milk TBS-Tween 20 for 1 h at room temperature. After three washings with TBS-Tween 20 (5 min each), the nitrocellulose membranes were further incubated with secondary antibodies diluted in Intercept® (TBS) Blocking Buffer (LI-COR Biosciences – GmbH) diluted ½ in TBS. To evaluate total protein staining, images of SyproRuby or Revert™ 700 -stained membranes were obtained by scanning on a Fusion FX (Vilber-Lourmat) or a Li-Cor Odyssey Infrared Imager (Immunoblots; Li-Cor

Biosciences, Lincoln, NE) respectively. All the images were digitalized and analyzed by Image Studio Lite Software (LI-COR Biosciences).

SUPPLEMENTARY FIGURES

Supplementary figure S1. Basic characterization of derived PGCs cultures. **a**, Expression of transcripts of germ cell and pluripotency markers in male and female chicken PGCs cultures (n=5) evaluated by RT QPCR. Relative transcript level was normalized by mean ratios of GAPDH and RPL15; * correspond to p<0.05. **b**, representative immunostaining of male and female PGCs cultures with the anti DDX4 (green) and SSEA1 (red) antibodies (ab). Nuclei are labelled with DAPI (blue). **c**, Colonisation of gonads of chicken embryos at 6,5 days of incubation by injecting in dorsal aorta of 52-57 hours embryos labelled with green fluorescent PKH67 dye PGCs providing from one male (at the top) and one female (at the bottom) PGCs cultures. Gonads (**g**), mesonephros (**m**), left (**L**), right (**R**). The gonads are delimited with white dotted line.

Supplementary figure S1a

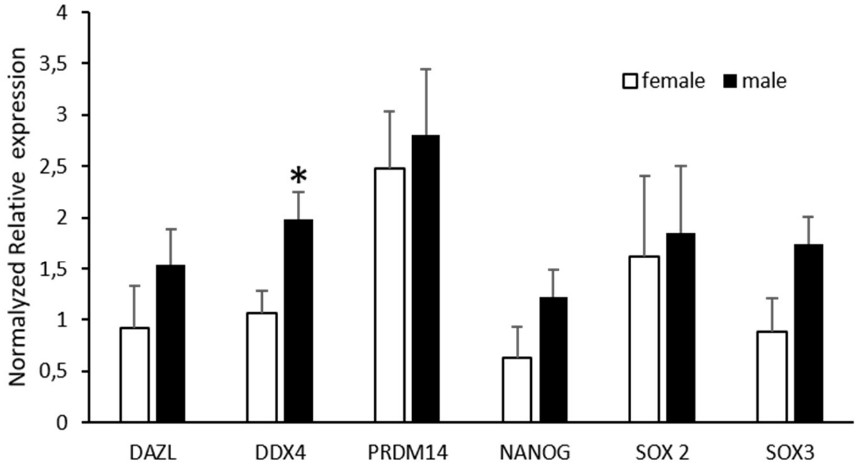

Supplementary figure S1b

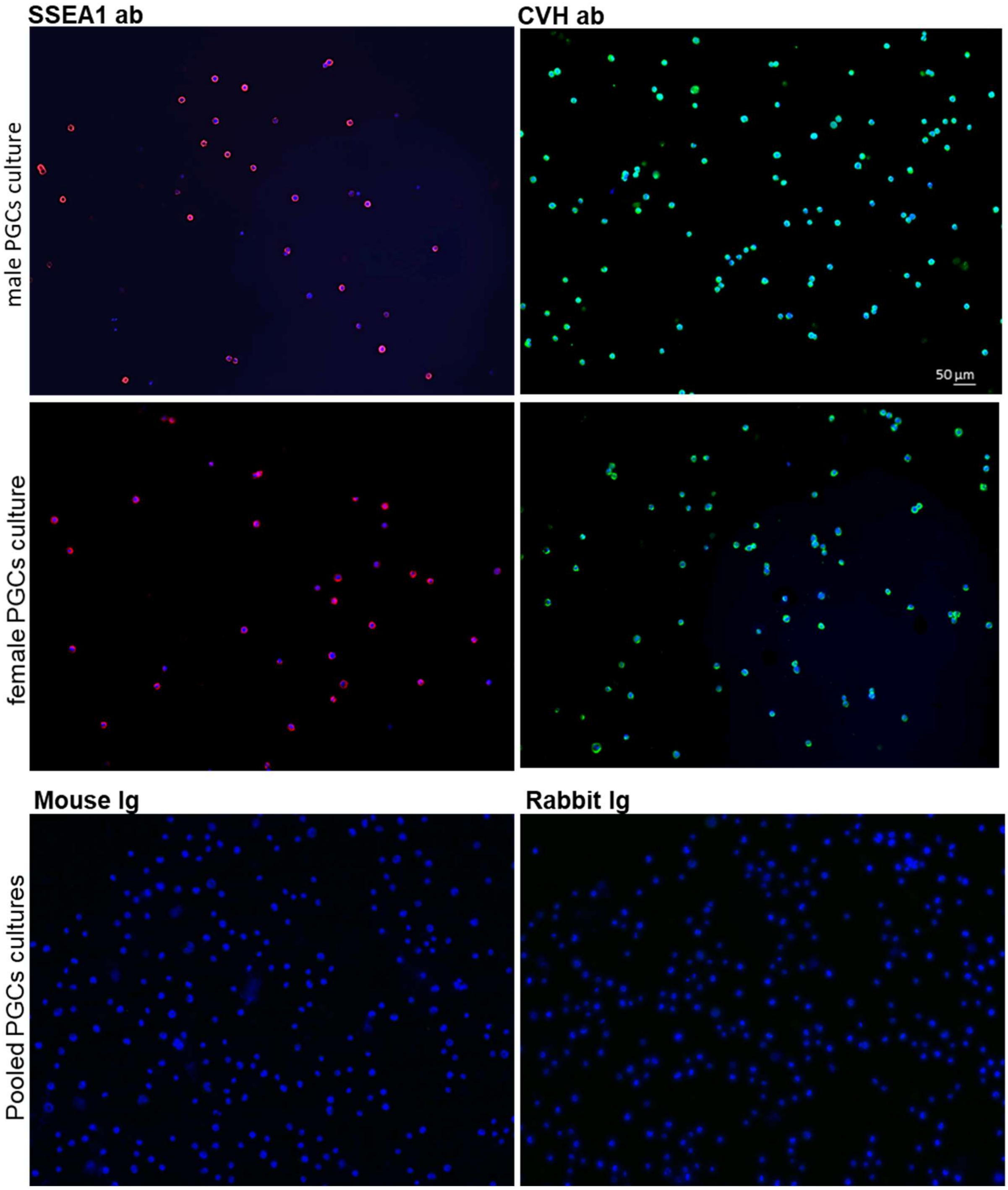

Supplementary figure S1c

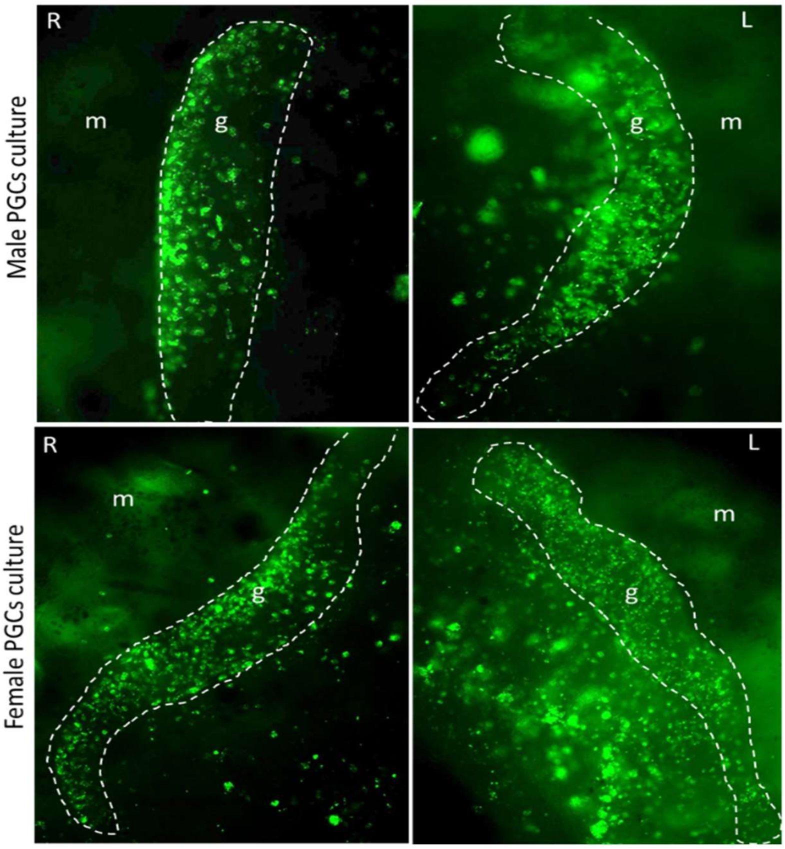

Supplementary figure S2. The pictures of the full-length nitrocellulose membranes with blotted proteins after total protein staining for IGF2BP1 analysis and after incubation with HSDL2 or IGF2BP1 antibodies.

Western blot analysis of HSDL2 and IGF2BP1 was performed on 5 individual female (ZW_{1-5}) and male (ZZ_{1-5}) PGCs cultures and mouse and chicken liver protein extracts as described in the section 2.9. The quantification of IGF2BP1and HSDL2 protein fluorescence is presented on **Fig1**. The bands corresponding to HSDL2 and IGF2BP1 molecular weights are indicated with arrows. White rectangles hide the parts of the blot that are not related to this work.

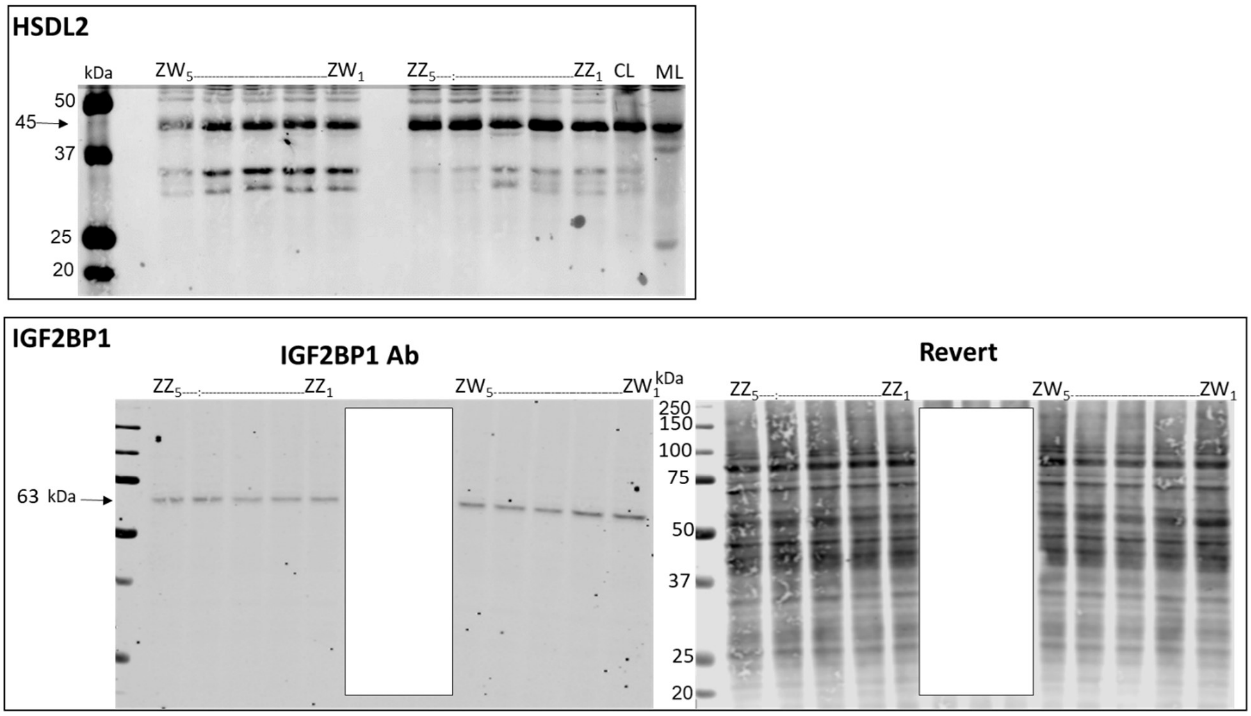

Supplementary figure S3. Western blot analysis of ovalbumin in male and female chicken PGCs.

Western blot analysis of ovalbumin was performed as described in the section 2.9 on the protein extracts of the pool of seven individual male (ZZ) PGCs cultures and of the pool of seven individual female (ZW) PGCs cultures different from those used for proteomic analysis. Three repetitions of WB experience were performed. The proteins were separated on 10% (WB1), 12% (WB2) or 15% (WB3) minigels. The band corresponding to the molecular weight of ovalbumin is indicated. Anti-ovalbumin antibodies (OVA ab), Total protein staining (Revert). White rectangles hide the parts of the membrane that are not related to this work. The results on the graph represent the means ± SEM for three experiences, P<0.01 (**).

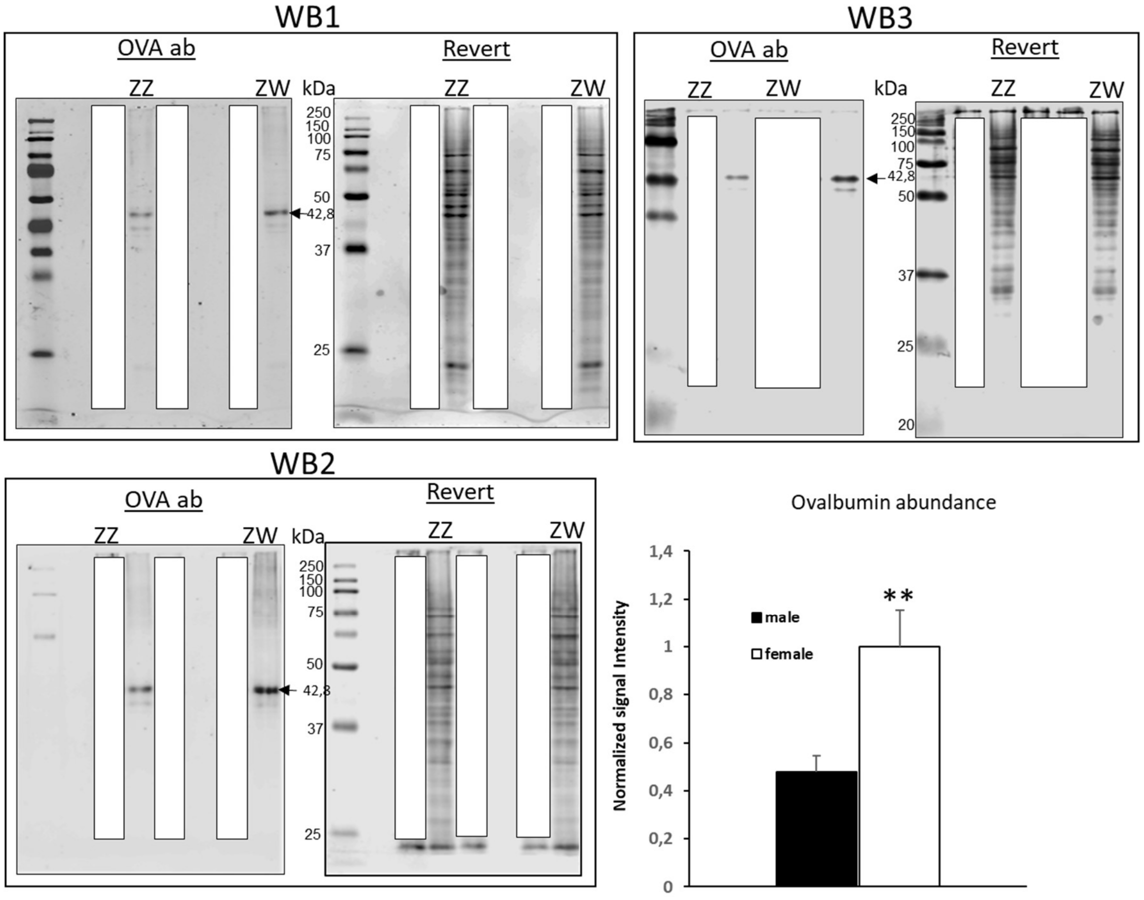

Supplementary figure S4. RT-QPCR analysis of genes encoding proteins overabundant in female PGC and identified in ovalbumin and chicken serum products.

One µg of total RNA extracted from the pools of male and female individual PGCs cultures and magnum was reverse transcribed and QPCR was performed with one µl of RT reaction diluted 1:20 for each sample. RT-QPCR was performed as described in the **METHODS.** Four reference genes (EEF1A, ACTB, GAPDH and RPL15) were used for sample comparison**.** Gene expression is presented as the mean of threshold cycle (**Ct**) of three replicates. The absence of bar means that the template was not detected after 45 QPCR cycles.

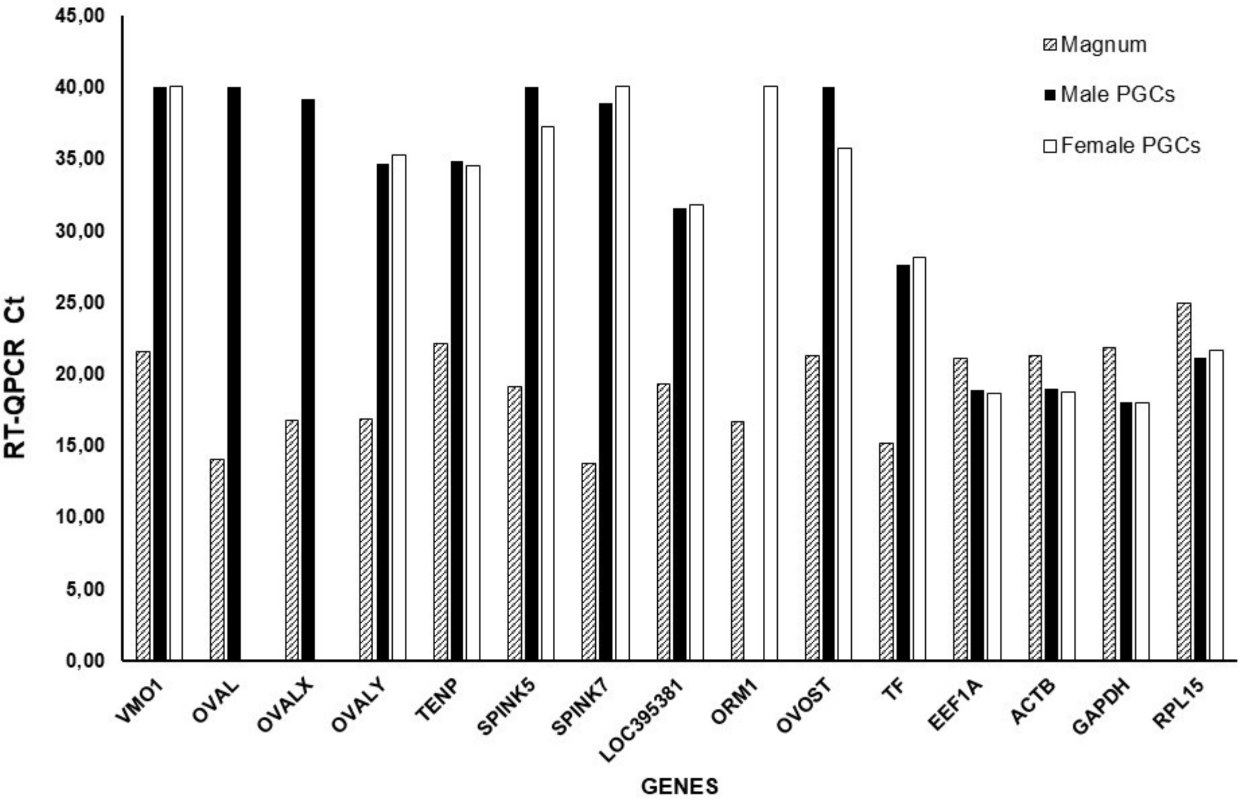

Supplement: Supplementary file 1 — Supplementary Information 1. [file 41598_2021_98454_MOESM1_ESM.pdf]
