# Supplementary material for: Protein expression reveals a molecular sexual identity of avian primordial germ cells at pre-gonadal stages
Source: Sci Rep. 2021 Sep 28;11:19236. doi: 10.1038/s41598-021-98454-2 (PMC8478952; doi:10.1038/s41598-021-98454-2)
Supplement: Supplementary file 2 — Supplementary Information 2. [file 41598_2021_98454_MOESM2_ESM.docx]

**Supplementary table S1.** Sequences of primers used for RT-QPCR.

| Gene Name | Gene ID | Forward Primer | Reverse Primer | NCBI Accession Number | Source |
| --- | --- | --- | --- | --- | --- |
| DAZL | 374054 | GAACGATGTCTGCAAATGCG | ATCCTTGGCAGGTTGTTGACG | NM_204218 | ^1^ |
| DDX4 | 395447 | TCTTGTGGCAACTTCGGTAGC | ACGACCAGTTCGTCCAATTCG | NM_204708 |  |
| NANOG | 100272166 | CAGCAGACCTCTCCTTGACC | TTCCTTGTCCCACTCTCACC | DQ867025.1 | ^2^ |
| PRDM14 | 100858709 | GAAATTCCCCTGCCACCTCT | GTGAACCCGCATGTGTTTGT | XM_015282907.1 |  |
| SOX2 | 396105 | CCAACTCGGCCGCGAACAAC | CGCGGGACCACACCATGAAG | NM_205188.2 | ^3^ |
| SOX3 | 374019 | TGTTCGCTTCCGAGTCTTAAA | CCTTTCCGTAGGAACAAAACC | NM_204195.1 | ^2^ |
| TLN1 | 374019 | GGAGGATGTCATCGCTACGG | GTGCTGGTGATCCTGCAGAA | NM_204523.1 |  |
| TTC37 | 427114 | CGAGGGGAGGTTACGTTGTT | GAGCTGTTGAGTGATCAGCAAC | XM_004949386.2 |  |
| ERMP1 | 426644 | ACTCCAGGTGCCAGTGATGA | ACGAGTGGGCCAAGTCAATT | XM_424271.5 |  |
| NUP210 | 415977 | GCCAATGGAGCTAACCCTGA | ACACCGTATGAATCTCTTCTACCAG | XM_414320.5 |  |
| WDR1 | 422842 | ACGAGGCGTGTCCAAAATCA | CCAGAGACATCTCCAGACGC | NM_001004402.1 |  |
| NUP155 | 427443 | TTGCAAAGGGGTCTTGCTGA | CAGATGACCACCCGTGTTGA | XM_004937197.2 |  |
| RPL30 | 425416 | ATGATTCGGCAAGGCAAAGC | AATGATGTCAGAGTCACCTGGG | NM_001007967.1 |  |
| ESIML1 | 418811 | TGCCCCCAATATCGAGCAAA | ACACCAAATCCACCAGGGAAA | NM_001282200.1 |  |
| PSMA7 | 395318 | TGTACCAGACTGACCCCTCT | CCAGACTGCACAACCTCAAGA | NM_204613.1 |  |
| H2AFY2 | 423721 | AGGCATTGTTCACCCCACAA | GTAAGTGCAGCTTCAGCAACT | NM_001277338.2 |  |
| SNRPA1 | 415523 | TCGACCTGCGGGGGTATAAA | GTCCTTCCCCAATCCGACAA | NM_001005823.1 |  |
| PPME1 | 419058 | GTTGGGAACGTGGTTGAAGC | CCATGGCTGTACCTTCCACA | NM_001030834.1 |  |
| IGF2BP1 | 395953 | GGTCAAGTCCGGCTATGCTT | AGCCATCCAGAACCTCCCAT | NM_205071.1 |  |
| SERPINH1 | 396228 | GCCATGTTCTTCAAGCCTCAC | ACCACCTGAAGCTTCTCTGC | NM_205291.1 |  |
| UCHL3 | 395626 | CGACGTCACCAATCAGTTTCTC | TACAGGTCTTGGCACCATGC | NM_204825.1 |  |
| COPS4 | 422594 | AGCTGACGGTTCCAGTATCC | TCATGCGCCCCTCAGTTATC | NM_001006447.1 |  |
| VMO1 | 418974 | GATTTGCACTGAAGGTTGAGC | AACTGGATGTTGTTGGCAGC | NM_001167761.1 |  |
| OVAL | 396058 | AAGACAGCACCAGGACACAGA | TTCTGGCAGATTGGGTATC | NM_205152.2 |  |
| OVALX | 420898 | TCCGTGAACATCCACCTACTCT | GGCTTGGTCTGATGCTGTTT | NM_001276386.1 |  |
| OVALY | 420897 | ACTGACTCTCAGTGTGGCTC | TGTAGCATTTGGCCTGGTGA | NM_001031001.1 |  |
| BPIFB2 | 395882 | GCAGCTCTGAAGCTCTTCCT | ATCCTCTATCACTGCCGTGGA | NM_205026.1 |  |
| SPINK5 | 416235 | TAAGGATGGCAGGACTTTGG | GAGTTTGCCACCAGTGGTTT | NM_001030612.2 |  |
| SPINK7 | 416236 | GCTGCTGTGAGTGTTGACTG | AGAGTCCCGTTGCTTTCCAC | XM_040646894.1 |  |
| LOC395381 | 395381 | CCACCTCATGGCAAGATGGA | ACAGTGGAGACCTGGACTATCT | NM_204661.1 |  |
| ORM1 | 395220 | GGTGTACATCATGGGTGCCT | CACGCATGTTTCATTCAGCCT | NM_204541.2 |  |
| OVOST | 396151 | TGACTACAGGGACACCCAGA | GCCCATGATGTCACCAACAAC | NM_205226.2 |  |
| TF | 396241 | AAGGCTCCACAACCAGCTAC | CTTGTTCCACTGAGCCCGAT | NM_205226.2 |  |
| EEF1A | 373963 | AGCAGACTTTGTGACCTTGCC | TGACATGAGACAGACGGTTGC | NM_001321516.1 |  |
| ACTB | 396526 | CAGATGTGGATCAGCAAGCAGG | TTTCATCACAGGGGTGTGGG | NM_205518.1 |  |
| GAPDH | 374193 | TGCTGCCCAGAACATCATCC | ATCAGCAGCAGCCTTCACTACC | NM_204305.1 |  |
| RPL15 | 428442 | TGTGATGCGTTTCCTCCTTCG | CCATAGGTTGCACCTTTTGGG | XM_015281569.3 |  |

1 Guibert, E. *et al.* Effects of mono-(2-ethylhexyl) phthalate (MEHP) on chicken germ cells cultured in vitro. *Environmental science and pollution research international* **20**, 2771-2783, doi:10.1007/s11356-013-1487-2 (2013).

2 Lavial, F. *et al.* The Oct4 homologue PouV and Nanog regulate pluripotency in chicken embryonic stem cells. *Development (Cambridge, England)* **134**, 3549-3563, doi:10.1242/dev.006569 (2007).

3 Jean, C. *et al.* Transcriptome analysis of chicken ES, blastodermal and germ cells reveals that chick ES cells are equivalent to mouse ES cells rather than EpiSC. *Stem cell research* **14**, 54-67, doi:10.1016/j.scr.2014.11.005 (2015).
